# Role of the medial prefrontal cortex and nucleus accumbens in an operant model of checking behaviour and uncertainty

**DOI:** 10.1177/2398212817733403

**Published:** 2017-09-28

**Authors:** Camilla d’Angelo, Dawn M. Eagle, Cristina-M. Coman, Trevor W. Robbins

**Affiliations:** 1 Department of Psychology, University of Cambridge, Cambridge, UK; 2 Behavioural and Clinical Neuroscience Institute (BCNI), University of Cambridge, Cambridge, UK; 3 Université Paris-Saclay, Saint-Aubin, France

**Keywords:** Striatum, prefrontal cortex, nucleus accumbens, checking, information-seeking, obsessive–compulsive disorder

## Abstract

**Background:**

Excessive checking is a common, debilitating symptom of obsessive–compulsive disorder. To further examine cognitive processes underpinning checking behaviour, and clarify how and why checking develops, we designed a novel operant paradigm for rats, the observing response task. The present study used the observing response task to investigate checking behaviour following excitotoxic lesions of the medial prefrontal cortex, nucleus accumbens core and dorsal striatum, brain regions considered to be of relevance to obsessive–compulsive disorder.

**Methods:**

In the observing response task, rats pressed an ‘observing’ lever for information (provided by light onset) about the location of an ‘active’ lever that provided food reinforcement. Following training, rats received excitotoxic lesions of the regions described above and performance was evaluated post-operatively before histological processing.

**Results:**

Medial prefrontal cortex lesions selectively increased functional checking with a less-prominent effect on non-functional checking and reduced discrimination accuracy during light information periods. Rats with nucleus accumbens core lesions made significantly more checking responses than sham-lesioned rats, including both functional and non-functional checking. Dorsal striatum lesions had no direct effect on checking per se, but reduced both active and inactive lever presses, and therefore changed the relative balance between checking responses and instrumental responses.

**Conclusions:**

These results suggest that the medial prefrontal cortex and nucleus accumbens core are important in the control of checking, perhaps via their role in processing uncertainty of reinforcement, and that dysfunction of these regions may therefore promote excessive checking behaviour, possibly relevant to obsessive-compulsive disorder.

## Introduction

Excessive checking is a common, debilitating symptom of obsessive–compulsive disorder (OCD) (Fontenelle et al., 2006). However, the neural mechanisms that underlie the development of excessive checking are not fully understood.

Functional neuroimaging and neuropsychological studies suggest that OCD is related to cortico-striatal hyperactivity (Graybiel and Rauch, 2000), particularly within the orbitofrontal-striatal circuitry (Menzies et al., 2008). Recent studies have implicated a wider network of regions in OCD pathology, including the nucleus accumbens (NAc), amygdala and the anterior cingulate cortical (ACC) region of the medial prefrontal cortex (mPFC) (Figee et al., 2011; Milad and Rauch, 2012). The NAc is clearly and directly implicated in OCD; OCD patients show altered ventral striatal activity when anticipating reward (Figee et al., 2011; Marsh et al., 2015; Remijnse et al., 2006) and effective deep-brain stimulation targets for treatment-refractory OCD patients are often in or around the ventral striatum (Greenberg et al., 2010). Although there is less direct evidence for ACC involvement in the OCD phenotype, ACC dysfunction is relevant to OCD development in a number of important ways. For example, ACC is involved in error detection and conflict monitoring (Van Veen and Carter, 2002) and electrophysiological and functional neuroimaging studies demonstrated increased error-related negativity (i.e. error detection) in OCD patients (Fitzgerald et al., 2005; Gehring et al., 2000; Ursu et al., 2003). Dysfunction of mPFC may also impair goal-directed control, leading to excessive habit formation, which may contribute to OCD symptoms (Gillan and Robbins, 2014). In rats, lesions to either the prelimbic cortex (PL), or its projection regions within the dorsomedial striatum, prevent acquisition of goal-directed learning and render performance habitual (Corbit and Balleine, 2003; Yin et al., 2005).

We recently established the observing response task (ORT) as a translational, operant test of checking behaviour, to examine the cognitive processes underpinning compulsive checking and its development (Eagle et al., 2014). In the ORT, rats can press a lever (the observing/checking response) that gives information about the location of future rewards. Uncertainty about which lever is reinforced can be reduced using the light-cue information provided by the ‘observing’ response; thus, checking can increase as a consequence of increased outcome uncertainty. Treatment with the dopamine D2/D3 receptor agonist quinpirole increased both ‘functional’ (for information) and also ‘non-functional’ (perseverative) checking in the ORT (Eagle et al., 2014). This quinpirole-induced checking is comparable with findings from a well-established open-field model of checking in rodents (Szechtman et al., 1998), in which excessive returns to a ‘home base’ in the open-field are interpreted as ‘checking behaviour’. Evidence from open-field checking also clearly implicates the NAc core in control of the vigour or extent of checking (Ballester González et al., 2015; Dvorkin et al., 2010). NAc core lesions increased ‘checking’ to an extent comparable with checking in unlesioned, quinpirole-treated rats (Dvorkin et al., 2010) and delayed, but did not prevent, the development of checking (Ballester González et al., 2015). Cortical input into the neural circuitry of checking is less clear; in contrast with NAc lesions, orbitofrontal cortex (OFC) lesions did not directly increase checking but produced changes in goal-directed activity in the open-field checking task. However, both the ACC and PL project to the NAc core (Brog et al., 1993; Vertes, 2004) and either may be a critical component of the cortico-striatal circuitry that normally moderates excessive checking. The role of these cortical structures to control checking behaviour remains to be investigated, alongside the role of their alternate striatal projection, the dorsal striatum (DStr).

NAc, DStr and mPFC are all potential sites-of-action for quinpirole in the ORT given their high concentration of D2 receptors. Subchronic quinpirole increased dopamine synthesis in the DStr and the NAc (Rowlett et al., 1995) but decreased dopamine levels in the mPFC (infralimbic cortex (IL), prelimbic cortex (PL) and ACC) (Sullivan et al., 1998). Similarly, there is reduced neuronal activity in the NAc core, DStr and mPFC of rats sensitised to quinpirole (Carpenter et al., 2003; Richards et al., 2005).

In the present study, we investigated the effects of inactivation of DStr, NAc core and mPFC, in ORT checking behaviour, using excitoxic (fibre-sparing) lesions of these structures, to determine the role of these regions in the neural circuitry underpinning control of checking behaviour.

## Materials and methods

### Subjects

Subjects were adult male Lister hooded rats sourced from Charles River, UK. Rats weighed 227 ± 8 g initially and 338 ± 21 g at the time of surgery (Experiment 1). Rats weighed 268 ± 2 g initially and 411 ± 5 g at the time of surgery (Experiment 2). Rats were housed in groups of four, in cages enriched with cardboard tubes, in a temperature-controlled room (minimum 22°C) under a reversed 12 h light-dark cycle (lights on 19:00; lights off 07:00). Rats were maintained at approximately 95% of their free-feeding weight and received 15–20 g of food daily (task reinforcer pellets plus laboratory chow given 1–2 h following the daily test session); this restricted weight gain to approximately 5 g per week. Water was available ad libitum for the duration of the procedures. All experiments were conducted in accordance with the United Kingdom Animals (Scientific Procedures) Act, 1986.

### Apparatus

**Figure 1.**
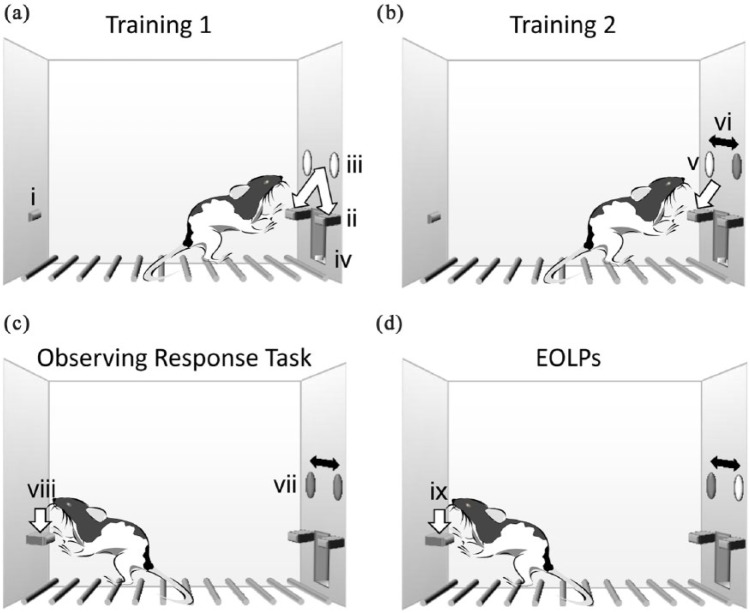
(a–d) The observing response task: (a) Training 1. The observing lever (i) on the back panel of the box was retracted. Rats were trained to press two levers (ii) on the front panel of the chamber. A light was illuminated above each lever to indicate ‘active’ status (iii). Completion of lever presses requirement gave a food pellet in a central food well (iv), (b) Training 2: lever discrimination. One front panel lever was active and the light above was illuminated (v); the other lever was inactive and the light above was unlit. The active lever and illuminated-light location switched on a pre-determined schedule (vi), (c) Observing response task. Both levers were extended but neither light was lit above (vii). The observing lever was extended, and a single press on the observing lever (viii) illuminated the light above the active lever for 15 s and (d) extra observing lever presses (EOLPs) (ix); when the active-lever light was illuminated, any further observing lever presses had no consequence, but were recorded as extra observing lever presses.

The apparatus and testing procedure have been described previously (Eagle et al., 2014). Testing took place in 12 operant-conditioning chambers (Med Associates). The chamber configuration is shown in Figure 1. Each chamber had two retractable levers, with a light above each, to the left and right of a central food well (Figure 1(a)). Illumination of the light above a lever signalled that the lever was currently active and delivered food pellets when pressed (Figure 1(b)). A third lever, the observing lever, was located in the centre panel of the back wall at the same height above the chamber floor as the active/inactive levers. If the observing lever was extended, a lever press turned on the light above the active lever if it was previously unlit (Figure 1(c)). A house light in the chamber roof was illuminated throughout the session. A pellet dispenser delivered 45 mg Noyes formula P pellets (TestDiet; Purina) into the food well when the active lever was pressed. Chamber operation and on-line data collection were controlled with the Observing Response Task program (written by A.C. Mar) and the Whisker server software (Cardinal and Aitken, 2010). Rats were tested between 5 and 7 days per week.

### Behavioural training

Rats received two training sessions per day for 15 training days (sessions 1–30), and one session per day during and subsequent to the last 3 days of observing lever training.

#### Training 1 – lever acquisition

Rats were trained to lever press for food pellets. Both front panel levers were presented and active (i.e. resulted in pellet delivery). The light above each lever was illuminated for the whole session. The observing lever remained retracted. Rats were reinforced with a food pellet on a fixed ratio (FR) 1 (sessions 1–2) or FR3 (session 3) schedule for completing the required presses on one or the other lever. Each session was terminated after 21 min or 200 rewards, whichever was sooner.

#### Training 2 – lever discrimination

Rats were trained to discriminate active from inactive levers. Both front panel levers were presented, one active and one inactive. The observing lever remained retracted. The light above the active lever was lit and the light above the inactive lever was unlit. The position of the active lever/light switched on a fixed time (FT) of 90 s schedule. The sequence always began with left lever active, which promoted more rapid learning; the rats could begin each session with predictably rewarded active lever location. Left and right levers were active for equal duration per session. An active lever press delivered food pellets on a pre-determined schedule of reinforcement (see below). Inactive lever pressing gave no consequence. If a rat switched from active to inactive lever, the active lever responses within a partially completed ratio were not reset to zero. However, the schedule requirement was restarted following a switch in location of active lever. Sessions ended after 21 min or 200 reward pellets. Rats were reinforced under the following schedules: FR3 increasing to FR10, variable ratio (VR) 5–15 increasing to VR10–20.

#### The observing response task

Rats were trained to make observing responses that ‘produce discriminative stimuli associated with the conditions of availability of primary reinforcement, but do not alter the availability of primary reinforcement’ (Wyckoff, 1952). At the beginning of the session, both front panel levers were presented, but there was no light illuminated above either lever. The observing lever was extended. One observing lever press (Figure 1(c)) illuminated the light above the currently active lever for a pre-determined period (30 s during training down to 15 s during the final version of the task). If the active lever switched location during the observing period, the light position switched correspondingly. While the active lever light was illuminated, any further observing lever presses had no consequence, but were recorded as extra observing lever presses (EOLPs; Figure 1(d)). EOLPs did not extend the period of light illumination. The active lever switched sides under an FT90s schedule. Rats were reinforced for active lever presses on a VR10–20 schedule. The session ended after 21 min or 200 reward pellets, whichever was sooner. Rats received nine sessions of observing lever training. Rats were trained twice a day (days 1–3; observing light duration of 30 s), decreasing to once a day (days 4–6; observing light duration of 15 s). A mean of sessions 31–33 constituted the pre-lesion baseline session.

### Behavioural challenges

Rats were challenged with manipulations of task contingencies in order to assess the effects of changing reward uncertainty on observing.

#### Unpredicted reward omission

Unpredicted reward omission (removal of expected reward) has previously been shown to significantly increase checking responses (Eagle et al., 2014). We tested the hypothesis that removal of reward/reinforcement pellets, when reward was expected, during a single reward omission session would increase checking. During reward omission, the session was identical to baseline (FT90s, VR10–20, OLP FR1 (15 s)), but the food reinforcer was delivered outside the test chamber (so all food delivery cues were identical except for food availability in the magazine).

#### Combined uncertainty of active lever location and contingency degradation

Because the effect of the mPFC lesion was small, we decided to test this group under an uncertainty schedule. We therefore tested the hypothesis that increased uncertainty about active lever location and response requirement would increase observing responses in this group. Uncertainty was increased by switching active/inactive lever location less predictably – from a FT90s to a variable time (VT) of 20–120 s schedule. For the contingency degradation, the response requirement changed from VR10–20 to variable interval (VI) of 10–20, and so behaviour became less linked to reinforcement. Thus, during reward uncertainty, the schedule was VT20-120s, VI10-20s, OLP FR1 (15 s). Combined unpredictability of active/inactive lever location (VT20–120s from VT90s) and effective contingency degradation (VI10–20s from VR10–20) was shown to significantly increase checking responses (d’Angelo PhD thesis, unpublished observations).

### Behavioural measures

The main measures on the ORT are detailed in the following:

*Active lever presses, light on (ALP on).* Responses on the active lever gave access to food pellets. Active lever presses completed when the light above the active lever was illuminated.
*Active lever presses, light off (ALP off).* Active lever presses completed when the light above the active lever was unlit.
*Inactive lever presses, light on (ILP on).* Responses on the inactive lever had no consequence. Inactive lever presses completed when the light above the active lever was illuminated.
*Inactive lever presses, light off (ILP off).* Inactive lever presses completed when the light above the active lever was unlit.
*Observing lever presses (OLPs).* Presses on the observing lever that turned on the active lever light.
*Extra observing lever presses (EOLPs).* Non-functional observing lever presses, completed during the period when the active lever light was illuminated, and that had no further consequence. These responses were perseverative, in the sense of being superfluous or non-functional, and could occur throughout the active lever light period.
*Rewards.* Total reward pellets per session.
*% Active light on.* % Active lever presses during the periods when the light above the active lever was lit, calculated as [100 × active/(active + inactive)]. *% Active light on* measured accuracy of responding on the active versus inactive lever during periods when the light gave information about which lever was currently active. We tested the hypothesis that rats were able to use the information from pressing the observing lever (i.e. turning on the light above the active lever) to locate, and therefore press, the active lever for food reward.
*% Active light off.* Calculated as in *% Active light on* above, but for the periods of the session when the light above the active lever was unlit.

### Surgery

#### Experiment 1

Following training, rats were allocated to three groups matched for baseline task performance of OLPs, EOLPs, active lever presses and rewards earned. Animals received lesions of the NAc core (n = 12), DStr (n = 12) or sham lesions (NAc core site, n = 6; DStr site, n = 6; total, n = 12). Rats were anaesthetised and secured in a stereotaxic frame (David Kopf Instruments). For all surgeries, the incisor bar was adjusted until the heights of lambda and bregma were equal so as to obtain a flat skull position. Rats were anaesthetised with inhaled isoflurane carried in medical oxygen, induced at 5% and maintained at 1%–4% concentrations at a flow rate of 2 L/min. Upon exposure of the skull, a dental drill was used to make small holes in the skull above the sites of microinjection. Lesion coordinates were derived using a stereotaxic atlas (Paxinos and Watson, 2005), using bregma as the origin. The dorsoventral reading was taken from dura. Animals were allowed up to 2 weeks to recover prior to behavioural re-training. For the first 24 h post-surgery, rats were singly housed and then returned to their pre-surgical groups. For 3 days post-surgery, rats received meloxicam analgesia in their drinking water (30 mg/L).

Excitotoxic lesions were made using 0.09 M quinolinic acid dissolved in 0.1 M phosphate-buffered saline (PBS; vehicle), with pH adjusted to 7.2–7.4 using 0.1 M NaOH. The toxin was infused using a 31-gauge stainless steel injector (Cooper’s Needle Works) connected via polyethylene tubing to a 10 μL glass Hamilton syringe (Hamilton Bonaduz AG) mounted on a microinfusion pump (Harvard Apparatus, Ltd.). Lesions were made according to parameters in Table 1. The injector was left in place at each site for a determined period following infusion in order to allow the diffusion of the toxin away from the injection site. Sham surgery was carried out in the same manner, except that vehicle was infused instead of toxin.

**Table 1.** Lesion coordinates used for lesions of the DStr, NAc core and mPFC.

Lesion	Sites/side	AP	ML	DV	Vol./site (μL)	Infusion time (min)	Diffusion time (min)
DStr	2	+0.2	±2.0	−5.0 −4.0	0.175	1:40	1:40
		+1.2	±2.0	−5.0 −4.0			
NAc core	1	+1.2	±1.8	−7.1	0.3	3	3
mPFC	3	+3.8	±0.6	−1.5	0.25	2	2
		+3.1	±0.6	−3.0	0.25	2	2
				−1.5	0.25		
		+2.4	±0.6	−1.5	0.25	2	2

DStr: dorsal striatum; NAc core: nucleus accumbens core; mPFC: medial prefrontal cortex; AP: anterior-posterior; ML: medial-lateral; DV:dorsoventral.

DV coordinates are from dura. Toxin infusion parameters: quinolinic acid, 0.09 M, via cannula.

#### Experiment 2

Following training, rats were allocated to two groups matched for pre-lesion task performance of OLPs, EOLPs, active lever presses and rewards earned. Animals received lesions of the mPFC (n = 16) or sham lesions (n = 14). The apparatus and procedures were similar to those in Experiment 1. Lesions were made according to parameters in Table 1.

Post-operatively, rats with sham lesions to each of the two different lesion sites were compared to assess whether they could be treated as one group for further analysis. Within these control groups, there was no evidence that the site of vehicle infusion had any effect on the primary measures of ORT performance. The NAc core group made more EOLPs than the DStr group pre-operatively (*F*_1,9_ = 5.698, *p* = .044). However, there were no significant pre-operative differences in any other behavioural measure (*p* > .05). Post-operatively, there were no significant differences between the control groups in any behavioural measure (group, *p* > .05). Sham-operated rats were therefore treated as one group for subsequent analyses.

### Histology

Rats were terminally anaesthetised with sodium pentobarbitone and perfused transcardially with 0.01 M PBS followed by formaldehyde solution (Experiment 1%–4% paraformaldehyde in PBS; Experiment 2%–10% neutral buffered formalin). Brains were removed, postfixed in the respective fixative and transferred into 20% sucrose in 0.01 M PBS before sectioning on a freezing microtome. Coronal sections (60 μm) were stained with cresyl violet and lesion locations were mapped onto standardised sections of the rat brain (Paxinos and Watson, 2005).

### Data analysis

Data were initially explored using box-plots and tests of homogeneity of variance so that outliers were identified and removed. Behavioural data were subjected to analysis of variance (ANOVA). A significance level of *p* < .05 was used for all analyses. Overall ANOVA was carried out on the data followed, where appropriate, by post hoc analysis. Analyses involving a single between-subjects factor and no within-subjects variables used the one-way ANOVA procedure. For repeated measures analyses, homogeneity of variance across groups was assessed by the Mauchly sphericity test and the degrees of freedom corrected to more conservative values using the Huynh–Feldt epsilon (Huynh, 1970) for any terms involving factors in which the sphericity assumption was violated. Significant main effects of interest were investigated further using pairwise comparisons with a Šidák correction. Where significant interactions were found, separate ANOVAs were conducted to establish simple effects.

## Results

### Histological analysis

Lesions were classified as acceptable if they showed significant damage or gliosis to the target area, with damage in both hemispheres, and no significant bilateral damage to the neighbouring structures.

#### Experiment 1

**Figure 2.**
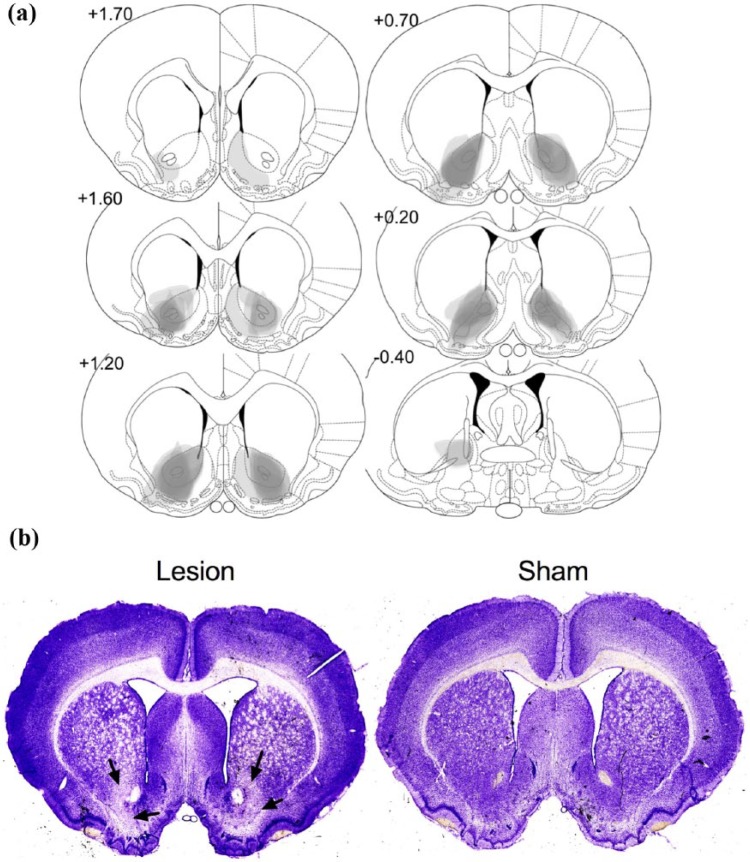
Histological analysis of NAc core lesions: (a) schematic diagrams of NAc core lesions. Grey scale shading indicates extent of neuronal loss across subjects, with each subject represented as a separate stacked layer. Diagrams are modified from Paxinos and Watson (2005) and (b) photomicrographs of cresyl-stained coronal sections, depicting typical lesions of NAc core-lesioned (left) and sham-lesioned (right) rats. Arrows indicate the site of lesion.

**Figure 3.**
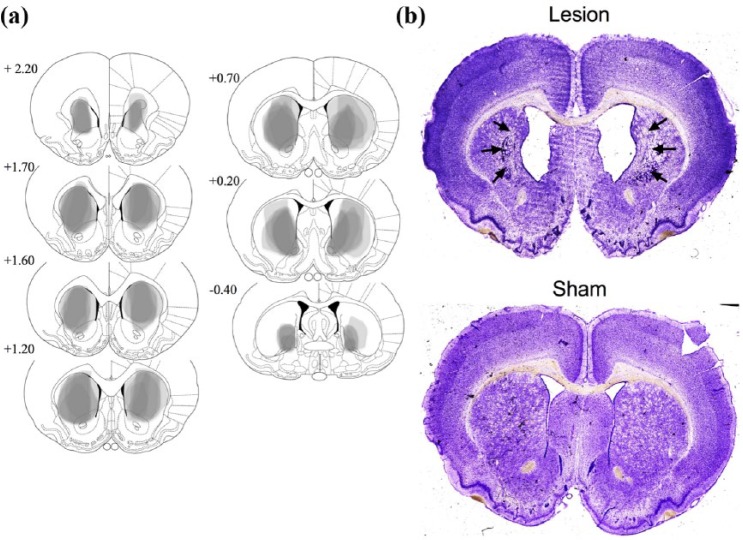
Histological analysis of DStr lesions: (a) schematic diagrams of DStr lesions. Grey scale shading indicates extent of neuronal loss across subjects, with each subject represented as a separate stacked layer. Diagrams are modified from Paxinos and Watson (2005) and (b) photomicrographs of cresyl-stained coronal sections, depicting typical lesions of DStr-lesioned (top) and sham-lesioned (bottom) rats. Arrows indicate the site of lesion.

Representative photomicrographs of both lesions and sham controls, and schematic representations of the extent of damage to the striatum caused by quinolinic acid infusions, are shown in Figure 2 (NAc core) and Figure 3 (DStr). (a) Five out of 12 rats with NAc core lesions were excluded as follows: Two rats had significant unilateral damage to the DStr. Three rats had significant damage to the NAc shell region, sparing most of the NAc core region. Seven of 12 NAc core lesion rats were determined to have appropriate lesions. The lesion started at approximately bregma +1.7 and extended to bregma +0.2. For all rats, the lesion encroached slightly into the lateral NAc shell. (b) Six of 12 DStr rats were excluded as the lesion extended bilaterally into the NAc core. Six of 12 DStr rats were determined to have appropriate lesions. The lesion started at approximately bregma +2.2 and extended to bregma –0.4. (c) One of 12 sham lesion rats was excluded due to extensive unilateral damage throughout the whole striatum. In all lesioned animals, there was considerable cell loss and gliosis in the lesioned regions, accompanied by striatal shrinkage. In DStr-lesioned rats, the extensive tissue shrinkage resulted in a visible widening of the lateral ventricles. One rat had baseline OLPs more than 2 standard deviations higher than the respective group mean OLPs and was removed from further analysis. Therefore, final group sizes for this experiment were Sham, n = 10; NAc core, n = 7; DStr, n = 6.

#### Experiment 2

**Figure 4.**
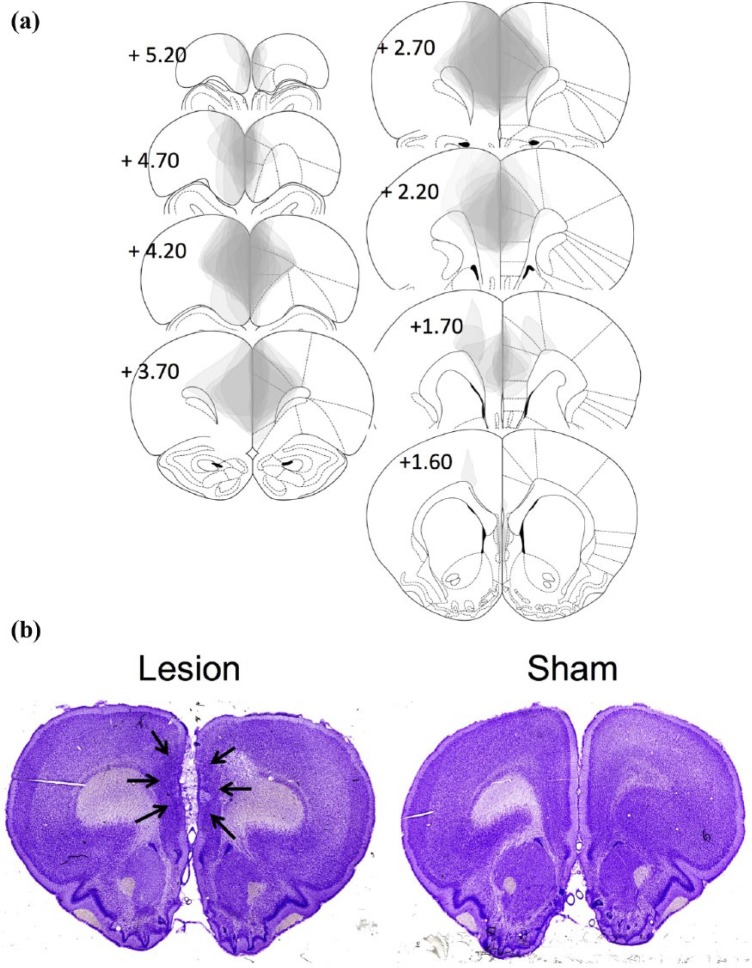
Histological analysis of mPFC lesions: (a) schematic diagrams of mPFC lesions. Grey scale shading indicates extent of neuronal loss across subjects, with each subject represented as a separate stacked layer. Diagrams are modified from Paxinos and Watson (28) and (b) photomicrographs of cresyl-stained coronal sections, depicting typical lesions of mPFC-lesioned (left) and sham-lesioned (right) rats. Arrows indicate the site of lesion.

Representative photomicrographs of both mPFC lesions and sham-lesioned controls and schematic representations of the extent of damage to the mPFC caused by quinolinic acid infusions are shown in Figure 4. Two of 16 rats with mPFC lesions were excluded because they had little or no apparent damage to the target regions of the mPFC. For all of the remaining rats in the mPFC group, each had significant damage to the anterior cingulate and prelimbic cortex, in both hemispheres. In two cases, there was minor encroachment of the lesion into medial orbital cortex. In six cases, there was minor unilateral encroachment of the lesion into the dorsal infralimbic cortex. There was no damage to the anterior parts of the striatum in any of the lesion group. For the control group, there was no evidence of lesion damage to the target area. Two rats (one lesion and one sham) had baseline OLPs more than 2 standard deviations higher than their respective group mean OLPs and were removed from further analysis. Therefore, final group sizes for this experiment were Sham, n = 13; mPFC, n = 13.

### Pre-surgical ORT performance

Pre-operatively, prospective lesion groups were matched for OLPs, EOLPs, total active lever presses and rewards earned. Following removal of subjects, there were no significant baseline differences in pre-operative performance between groups with respect to any measure (Table 2; all *F*s < 1; except Experiment 2: *ILP on, F*_1,25_ = 4.369, *p* = .047).

**Table 2.** Pre-surgical ORT performance.

Measure	Experiment 1			Experiment 2	
	Sham	NAc core	DStr	Sham	mPFC
OLPs	0.18 ± 0.04	0.22 ± 0.06	0.26 ± 0.08	0.15 ± 0.02	0.19 ± 0.05
EOLPs	0.13 ± 0.04	0.12 ± 0.04	0.23 ± 0.11	0.2 ± 0.05	0.28 ± 0.07
ALP on	24.89 ± 6.2	26.4 ± 6.15	23.56 ± 6.81	36.69 ± 7.99	32.13 ± 5.41
ALP off	64 ± 4.8	59.59 ± 7.68	57.69 ± 7.38	63.86 ± 6.23	68.8 ± 6.2
ILP on	5.34 ± 1.52	8.17 ± 3.55	11.34 ± 3.91	9.12 ± 1.42	14.36 ± 2.07
ILP off	20.92 ± 0.81	21.53 ± 2.33	21.17 ± 1.45	22.24 ± 1.08	22.46 ± 1.43
Rewards	3.96 ± 0.31	3.7 ± 0.47	3.48 ± 0.4	4.03 ± 0.41	4.28 ± 0.4
% Active on	61.5 ± 10.05	73.3 ± 6.65	72.46 ± 7.88	66.05 ± 6.98	59.99 ± 7.1
% Active off	74.64 ± 1.63	71.78 ± 3.57	72.14 ± 2.83	72.32 ± 2.52	73.66 ± 2.61

NAc core: nucleus accumbens core; DStr: dorsal striatum; mPFC: medial prefrontal cortex; OLPs: observing lever presses; EOLPs: extra observing lever presses; ALP on: active lever presses, light on; ALP off: active lever presses, light off; ILP on: inactive lever presses, light on; ILP off: inactive lever presses, light off; Experiment 1: lesions of the NAc core, DStr and controls. Experiment 2: lesions of the mPFC and controls.

Data are expressed as mean ± SEM.

### Post-surgical ORT performance

Following recovery from surgery, rats were tested for 20 daily sessions under the standard schedule of the task (FT90s, VR10-20, OLP FR1 (15 s)).

#### Effects on checking

##### Functional checking (OLPs)

**Figure 5.**
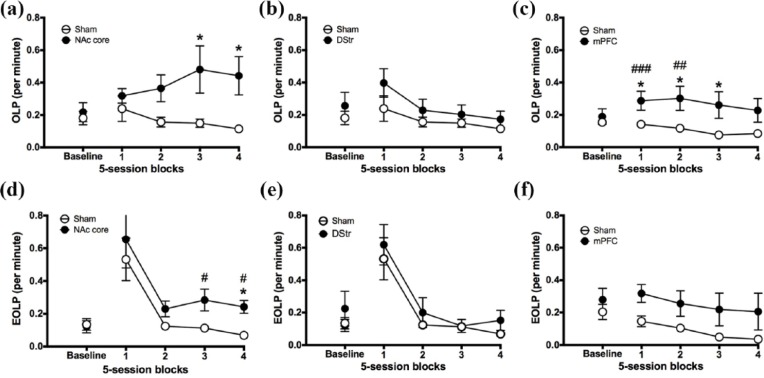
The effect of NAc core, DStr and mPFC lesions on checking. Figures show pre-surgery baseline sessions (three sessions) and 20 post-surgical sessions for NAc core (left), DStr lesion (middle) and mPFC (right) rats and sham-operated controls. (a, b, c) functional OLPs; (d, e, f) non-functional EOLPs. Significance is denoted as follows: #*p* < 0.05, ##*p* < 0.01 versus baseline in the lesion groups; *p < 0.05 between groups.

*Experiment 1.* Figure 5(a) shows that NAc core lesions significantly increased functional checking, in particular during the final 10 sessions post-lesion (pre-post block × lesion, group × block: *F*_2.188,32.824_ = 4.706, *p* = .014; pre-post block, block 3: *F*_1,15_ = 7.781, *p* = .014; block 4: *F*_1,15_ = 9.619, *p* = .007). During this time, rats with NAc core lesions made significantly more OLPs than controls (group, block 3: *F*_1,15_ = 4.63, *p* = .048; block 4: *F*_1,15_ = 6.392, *p* = .023). In contrast, control rats significantly reduced their rate of checking relative to pre-lesion baseline during the last five sessions (pre-post block × lesion, block 4: *F*_1,9_ = 5.739, *p* = .04). Figure 5(b) shows that there was no effect of DStr lesions on functional checking (pre-post block × lesion, block 1: *F*_1,14_ = 1.601, *p* = .226; blocks 2–4: *F* < 1).

*Experiment 2.* Figure 5(c) shows that mPFC lesions significantly increased functional checking, in particular during the first 10 sessions post-lesion (pre-post block × lesion, block × lesion: *F*_2.868,68.821_ = 4.112, *p* = .011; block 1: *F*_1,24_ = 14.653, *p* = .001; block 2: *F*_1,24_ = 12.536, *p* = .002). During this time, rats with mPFC lesions made significantly more OLPs relative to pre-lesion baseline (pre vs post block 1: *F*_1,12_ = 18.996, *p* = .001; block 2: *F*_1,12_ = 9.184, *p* = .01), whereas control rats did not (pre vs post block, 1: *F* < 1; block 2: *F*_1,12_ = 3.39, *p* = .09, Not Significant (NS)). Rats with mPFC lesions also made significantly more OLPs than controls (group, block 1: *F*_1,25_ = 5.268, *p* = .031; block 2: *F*_1,25_ = 5.652, *p* = .026; block 3: *F*_1,25_ = 4.913, *p* = .036; block 4: *F*_1,25_ = 3.567, *p* = .071, NS). mPFC rats tended to reduce checking across time compared with immediate post-surgery effects (pre-post block × lesion, block 3: *F*_1,12_ = 2.342, *p* = .152, NS; block 4: *F* < 1). Control rats also reduced their rate of checking relative to pre-lesion during the last two blocks (pre-post block × lesion, block 3: *F*_1,12_ = 27.38, *p* = .0002; block 4: *F*_1,12_ = 23.567, *p* = .0003).

##### Non-functional checking (EOLPs)

*Experiment 1.* The finding that EOLPs transiently increased after surgery for both groups suggests that rats may not have fully recovered from surgery, and that this was an effect of inflammation. Thus, any effects of lesion may only be evident later on in training, when EOLPs have had time to stabilise. When sessions 1–5 are excluded from analysis, Figure 5(d) shows that NAc core lesions significantly increased non-functional checking, in particular during the final 10 sessions post-lesion (pre-post block × lesion, *F*_3,45_ = 4.765, *p* = .006; pre-post block, block 3: *F*_1,15_ = 8.736, *p* = .01; block 4: *F*_1,15_ = 11.335, *p* = .004). During this time, rats with NAc core lesions made significantly more EOLPs relative to pre-lesion baseline (pre vs post, block 3: *F*_1,6_ = 11.24, *p* = .015; block 4: *F*_1,6_ = 8.612, *p* = .026), whereas control rats did not (pre vs post, block 3: *F* < 1; block 4: *F*_1,9_ = 3.282, *p* = .103, NS). During the last five sessions, rats with NAc core lesions also made significantly more EOLPs than controls (group, *F*_1,15_ = 4.837, *p* = .044). As seen in Figure 5(e), there was no effect of DStr lesion on non-functional checking (pre-post block × lesion, block × lesion *F* < 1; block, *F*_1.996,27.938_ = 1.972, *p* = .158, NS).

*Experiment 2.* Figure 5(f) shows that mPFC lesions had no significant effect on non-functional checking (pre-post block × lesion, block × lesion: *F* < 1, NS). However, there was a significant main effect of block (*F*_1.847,44.329_ = 3.546, *p* = .041). Further analysis revealed that during the last 10 sessions, all rats reduced their rate of EOLPs relative to pre-lesion baseline (pre vs post, block 3: *F*_1,24_ = 4.296, *p* = .049; block 4: *F*_1,24_ = 4.471, *p* = .045).

#### Effects on instrumental responding

##### Experiment 1

**Figure 6.**
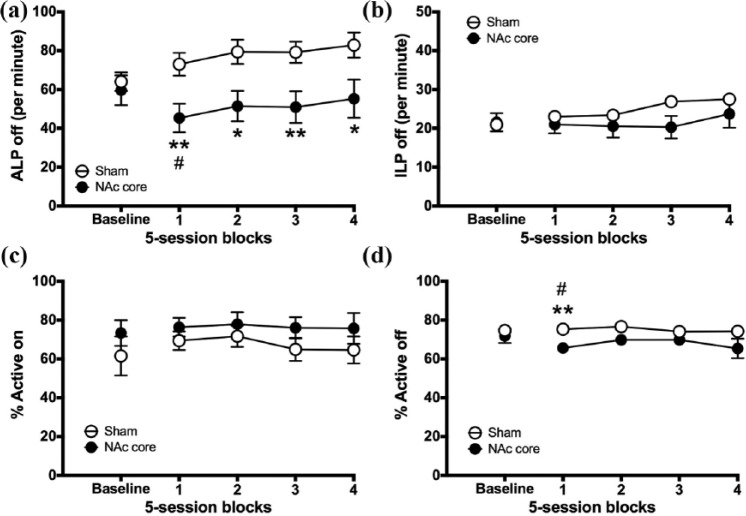
The effect of NAc core lesions on instrumental responding. Figure shows pre-surgery baseline (three sessions) and 20 post-surgical sessions for NAc core rats and sham-operated controls: (a) ALP off, (b) ILP off, (c) % Active on and (d) % Active off. Significance is denoted as follows: #*p* < 0.05, versus baseline in the NAc core group; *p < 0.05, **p < 0.01 between groups.

**Figure 7.**
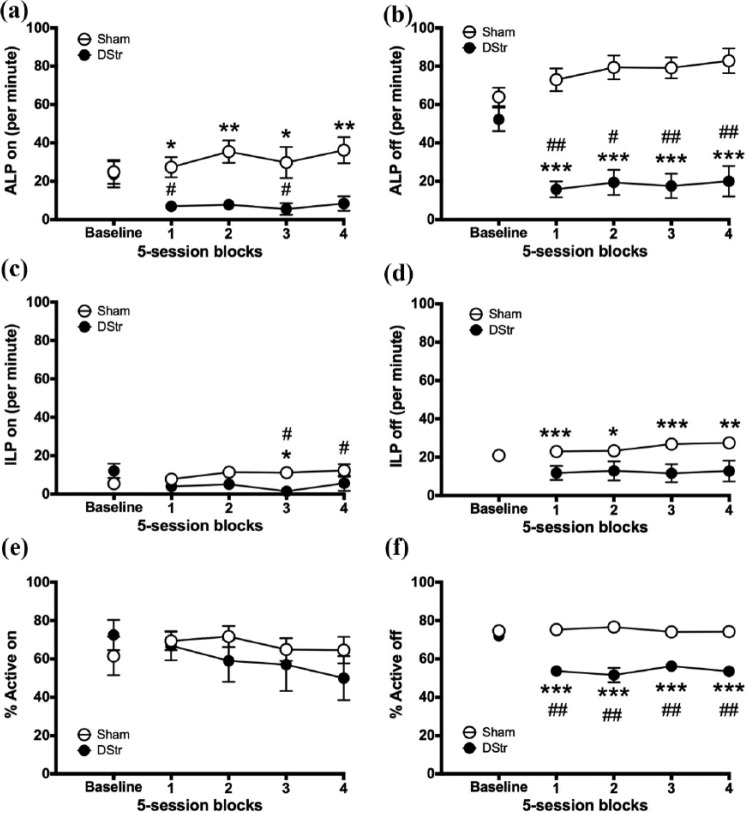
The effect of DStr lesions on instrumental responding. Figures show pre-surgery baseline sessions (three sessions) and 20 post-surgical sessions for DStr lesion rats and sham-operated controls: (a) ALP on, (b) ALP off, (c) ILP on, (d) ILP off, (e) % Active on and (f) % Active off. Significance is denoted as follows: #*p* < 0.05, ##*p* < 0.01 versus baseline in the DStr group; *p < 0.05, **p < 0.01, ***p < 0.001 between groups.

- Figure 6(a) shows that NAc core lesions significantly reduced the rate of active lever presses during periods when the active lever light was unlit (pre-post block × lesion, block × lesion: *F*_2.852,42.784_ = 6.52, *p* = .001; block 1: *F*_1,15_ = 20.92, *p* = .000365; block 2: *F*_1,15_ = 19.736, *p* = .000475; block 3: *F*_1,15_ = 18.28, *p* = .001; block 4: *F*_1,15_ = 14.958, *p* = .002). During this time, rats with NAc core lesions made fewer active lever presses relative to pre-lesion baseline; however, this was only significant during the first block (pre vs post, block 1: *F*_1,6_ = 8.715, *p* = .026; block 2: *F*_1,6_ = 3.024, *p* = .133, NS; block 3: *F*_1,6_ = 4.094, *p* = .089, NS; block 4: *F* < 1). By contrast, control rats increased their rate of active lever presses relative to pre-lesion baseline (pre vs post, block 1: *F*_1,9_ = 11.808, *p* = .007; block 2: *F*_1,9_ = 26.547, *p* = .001; block 3: *F*_1,9_ = 18.040, *p* = .002; block 4: *F*_1,9_ = 28.228, *p* = .000485). During this period, rats with NAc core lesions made significantly fewer active lever presses than controls (group, block 1: *F*_1,16_ = 8.727, *p* = .01; block 2: *F*_1,16_ = 7.939, *p* = .013; block 3: *F*_1,16_ = 9.004, *p* = .009; block 4: *F*_1,16_ = 5.966, *p* = .027). This resulted in a reduction in task accuracy during periods when the light was unlit (% Active off) during the first block (pre-post block × lesion, *F*_1,15_ = 9.364, *p* = .008) (Figure 6(d)). During this time, rats with NAc core lesions reduced their accuracy relative to pre-lesion baseline (pre vs post, block 1, *F*_1,6_ = 8.05, *p* = .03) and were less accurate relative to controls (group, *F*_1,16_ = 9.05, *p* = .009). There was no effect of NAc core lesion on any other measure of instrumental responding (pre-post block × lesion, all measures, *F* < 1; Figure 6(b) and (c), Figure S1 and for full analyses see Table S1), except for a slight reduction in the rate of inactive lever presses during periods without information but only during block 3 (Figure 6(b)).
- Figure 7 indicates that DStr lesions led to a significant impairment in instrumental responding, both when the active lever light was lit and when it was unlit.

#### Periods when the active lever light was lit

Figure 7(a) indicates that DStr lesions reduced the rate of active lever presses during periods when the active lever light was lit (pre-post block × lesion, block 1: *F*_1,14_ = 5.967, *p* = .028; block 2: *F*_1,14_ = 10.256, *p* = .006; block 3: *F*_1,14_ = 6.202, *p* = .026; block 4: *F*_1,14_ = 10.47, *p* = .006). During the post-surgical period, rats with DStr lesions made fewer active lever presses relative to pre-lesion baseline, and this was significant during block 1 (pre vs post, *F*_1,5_ = 9.486, *p* = .027) and block 3 (pre vs post, *F*_1,5_ = 11.66, *p* = .019). In contrast, by block 4, control rats increased their rate of active lever presses relative to pre-lesion baseline (pre vs post, *F*_1,9_ = 6.548, *p* = .031). During this period, rats with DStr lesions made significantly fewer active lever presses than controls (group, block 1: *F*_1,15_ = 8.122, *p* = .013; block 2: *F*_1,15_ = 12.274, *p* = .004; block 3: *F*_1,15_ = 4.928, *p* = .043; block 4: *F*_1,15_ = 8.865, *p* = .01). Despite reducing their rate of active lever presses, rats with DStr lesions were not statistically significantly impaired in discrimination accuracy during periods of light illumination relative to controls (pre-post block × lesion, all *p* > .05) (Figure 7(e)). However, by block 4, their discrimination approximated 50%, suggesting that they were not discriminating between levers.

Although rats with DStr lesions reduced their rate of inactive lever presses relative to pre-lesion baseline during periods when the active lever light was lit, this was not statistically significant (Figure 7 (c); pre vs post, all blocks *p* > .05). During block 3, however, DStr rats made fewer inactive lever presses relative to controls (group, *F*_1,15_ = 9.832, *p* = .007). In contrast, by blocks 3 and 4, control rats increased their rate of inactive lever presses relative to pre-lesion baseline (pre vs post, block 3: *F*_1,9_ = 13.895, *p* = .005; block 4: *F*_1,8_ = 11.948, *p* = .009).

#### Periods when the active lever light was unlit

Figure 7(b) indicates that DStr lesions reduced the rate of active lever presses during periods when the active lever light was unlit (pre-post block × lesion, block 1: *F*_1,14_ = 46.385, *p* < .0001; block 2: *F*_1,14_ = 41.426, *p* < .0001; block 3: *F*_1,14_ = 45.313, *p* < .0001; block 4: *F*_1,14_ = 47.452, *p* < .0001). During the post-surgical period, rats with DStr lesions made fewer active lever presses relative to pre-lesion baseline (pre vs post, block 1: *F*_1,5_ = 22.658, *p* = .005; block 2: *F*_1,5_ = 15.667, *p* = .011; block 3: *F*_1,5_ = 20.011, *p* = .007; block 4: *F*_1,5_ = 17.818, *p* = .008). By contrast, control rats increased their rate of active lever presses relative to pre-lesion baseline (pre vs post, block 1: *F*_1,9_ = 11.808, *p* = .007; block 2: *F*_1,9_ = 26.547, *p* = .001; block 3: *F*_1,9_ = 18.04, *p* = .002; block 4: *F*_1,9_ = 28.228, *p* = .001). Furthermore, during this period, rats with DStr lesions made significantly fewer active lever presses than controls (group, block 1: *F*_1,15_ = 50.921, *p* < .0001; block 2: *F*_1,15_ = 44.932, *p* < .0001; block 3: *F*_1,15_ = 58.068, *p* < .0001; block 4: *F*_1,15_ = 42.51, *p* < .0001).

Similarly, Figure 7(d) shows that DStr lesions reduced the rate of inactive lever presses during periods when the active lever light was unlit (pre-post block × lesion, block 1: *F*_1,14_ = 58.33, *p* = .012; block 2: *F*_1,14_ = 4.949, *p* = .043; block 3: *F*_1,14_ = 11.335, *p* = .005; block 4: *F*_1,14_ = 7.99, *p* = .013). Although DStr rats showed no change in the rate of inactive lever presses relative to pre-lesion baseline (pre vs post, block 1: *F*_1,5_ = 3.583, *p* = .117; block 2: *F*_1,5_ = 1.84, *p* = .233; block 3: *F*_1,5_ = 2.744, *p* = .159; block 4: *F*_1,5_ = 1.629, *p* = .258, all NS), control rats increased their rate of inactive lever presses relative to pre-lesion baseline (pre vs post, block 1: *F*_1,9_ = 4.893, *p* = .054, NS; block 2: *F*_1,9_ = 5.454, *p* = .044; block 3: *F*_1,9_ = 28.173, *p* = .001; block 4: *F*_1,9_ = 22.579, *p* = .001). Furthermore, during this period, rats with DStr lesions made significantly fewer inactive lever presses than controls (group, block 1: *F*_1,15_ = 17.462, *p* = .001; block 2: *F*_1,15_ = 6.974, *p* = .019; block 3: *F*_1,15_ = 17.462, *p* = .001; block 4: *F*_1,15_ = 10.885, *p* = .005).

Figure 7(f) shows that during periods when the light was unlit, the impairment in instrumental responding led to an impairment in discrimination accuracy (pre-post block × lesion, block 1: *F*_1,14_ = 8.438, *p* < .0001; block 2: *F*_1,14_ = 75.701, *p* < .0001; block 3: *F*_1,14_ = 81.991, *p* < .0001; block 4: *F*_1,14_ = 32.08, *p* < .0001). During the post-surgical period, rats with DStr lesions were less accurate relative to pre-lesion baseline (pre vs post, block 1: *F*_1,5_ = 46.036, *p* = .001; block 2: *F*_1,5_ = 52.259, *p* = .001; block 3: *F*_1,5_ = 118.61, *p* = .001; block 4: *F*_1,5_ = 39.96, *p* = .001). Furthermore, during this period, rats with DStr lesions were significantly less accurate than controls (group, block 1: *F*_1,15_ = 72.168, *p* < .0001; block 2: *F*_1,15_ = 50.502, *p* < .0001; block 3: *F*_1,15_ = 60.48, *p* < .0001; block 4: *F*_1,15_ = 28.173, *p* < .0001). Control rats showed no change in discrimination accuracy relative to pre-lesion baseline (pre vs post, block 1: *F* < 1; block 2: *F*_1,9_ = 3.188, *p* = .108, NS; block 3: *F* < 1; block 4: *F* < 1).

##### Experiment 2

**Figure 8.**
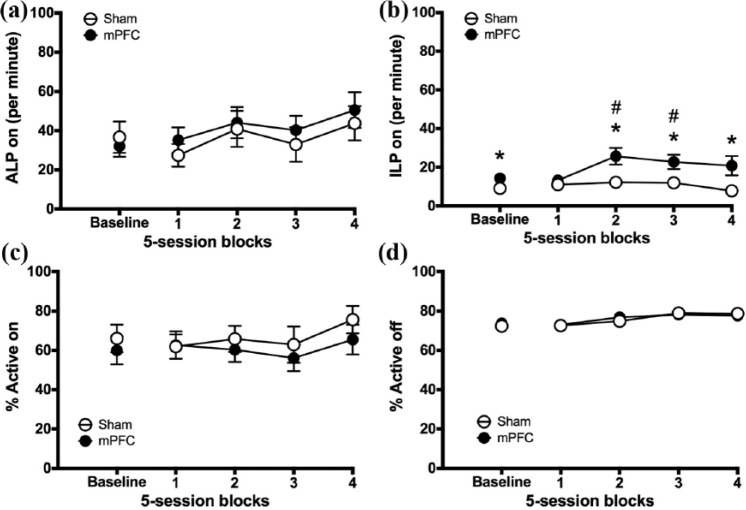
The effect of mPFC lesions on instrumental responding. Figure shows pre-surgery baseline sessions (three sessions) and 20 post-surgical sessions for mPFC lesion rats and sham-operated controls: (a) ALP on, (b) ILP on, (c) % Active on and (d) % Active off. Significance is denoted as follows: #*p* < 0.05 versus baseline in the mPFC group; *p < 0.05 between groups.

Figure 8(b) shows that mPFC lesions increased the rate of inactive lever presses, but only during periods when the active lever light was lit (pre-post block × lesion, block × lesion: *F*_3.357,77.207_ = 2.858, *p* = .037; block 1: *F*_1,24_ = 1.037, *p* = .319, NS; block 2: *F*_1,24_ = 7.105, *p* = .014; block 3: *F*_1,24_ = 5.394, *p* = .029; block 4: *F*_1,23_ = 1.707, *p* = .204, NS). During blocks 2 and 3, rats with mPFC lesions made significantly more inactive lever presses relative to pre-lesion baseline (pre vs post, block 2: *F*_1,12_ = 6.848, *p* = .023; block 3: *F*_1,12_ = 5.214, *p* = .041). During the last three blocks, rats with mPFC lesions also made more inactive lever presses than controls (group, block 2: *F*_1,25_ = 6.782, *p* = .016; block 3: *F*_1,25_ = 5.373, *p* = .029; block 4: *F*_1,24_ = 5.736, *p* = .025). However, Figure 8(c) shows that this did not result in a significant reduction in task accuracy (% Active on) (pre-post block × lesion, block × lesion: *F* < 1, NS; block: *F*_3.172,69.795_ = 1.522, *p* = .215, NS). Indeed, comparison of the percentage of active versus inactive lever presses revealed that all groups made a greater percentage of active lever presses compared with inactive lever presses (both when the light was lit and unlit) during pre-lesion baseline and each of the post-surgery blocks (all blocks, *p *≤ .05; Figure S5; Table S5). There was no effect of mPFC lesion on any other measure of instrumental responding (pre-post block × lesion, all measures, *F* < 1; Figure 8(a) and (d), Figure S4 and for full analyses, see Table S4).

### Performance during unpredicted reinforcer omission

Following post-surgical testing, rats completed one omission-of-reinforcer session and five (Experiment 1) or three (Experiment 2) recovery baseline sessions under the standard schedule of the task (see Supplementary Material).

### Performance during uncertainty

*Experiment 2.* Following three recovery baseline sessions under the standard schedule of the task, rats were tested for 25 daily sessions under the following schedule: VT20-120s, VI10-20, OLP FR1 (15 s). Uncertainty blocks were compared with the data from the three recovery baseline sessions, which served as the new baseline for the following analyses.

#### Effects on checking

##### Functional checking (OLPs)

**Figure 9.**
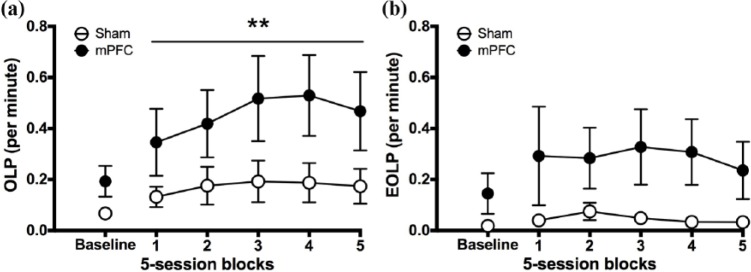
Effects of uncertainty on checking. Figures show baseline sessions (three sessions under the standard schedule of the task) and 25 sessions of uncertainty for mPFC lesion rats and sham-operated controls: (a) OLPs and (b) EOLPs. Significance is denoted as follows: **p < 0.01 between Baseline and the mean of Blocks 1–5 (Block effect).

Figure 9(a) shows that the uncertainty manipulation increased OLP irrespective of lesion group (pre-post block × lesion, block, *F*_5,120_ = 7.835, *p* = .0001; block × group, *F*_2.174,52.186_ = 1.68, *p* = .199, NS; group, *F*_1,24_ = 3.299, *p* = .082, NS). Post hoc analysis compared baseline to the average of blocks 1 to 5, irrespective of group, and showed a significant difference (pre-post block, *F*_1,29_ = 10.719, *p* = .003).

However, as evident in Figure 9(a), there was a strong trend for the mPFC lesion group to exhibit more OLP than the Shams, as had been shown previously (Figure 5(c)).

##### Non-functional checking (EOLPs)

Figure 9(b) shows that there was no effect of uncertainty on the rate of non-functional checking in either group (pre-post block × lesion, block, *F*_2.727,65.458_ = 1.625, *p* = .196, NS; block × group, *F* < 1, NS; group, *F*_1,24_ = 3.282, *p* = .083, NS).

#### Effects on instrumental responding

Figure S6 shows that when reinforcement was made more uncertain, there was no effect of uncertainty on any measure of instrumental responding (pre-post block × lesion, all measures, *F* < 1) (for full analyses, see Table S6). Figure S6(c) shows that rats with mPFC lesions made more inactive lever presses than controls during periods of light illumination (pre-post block × lesion, group: *F*_1,18_ = 113.58, *p* = .0001; block 1: *F*_1,24_ = 7.966, *p* = .01; block 2: *F*_1,24_ = 10.341, *p* = .004; block 3: *F*_1,24_ = 5.144, *p* = .033; block 4: *F*_1,23_ = 12.567, *p* = .002; block 5: *F*_1,21_ = 11.342, *p* = .003); however, this was not a selective effect of uncertainty (pre-post block × lesion, block × lesion: *F* < 1, NS).

### Summary of main findings

**Table 3.** Summary of behavioural effects of NAc core, DStr and mPFC lesions on the ORT during all experimental challenges.

	Post-operative baseline			Uncertainty
	NAc core	DStr	mPFC	mPFC
OLPs	↑	-	↑	+
EOLPs	↑	-	↑	-
ALP on	-	↓	-	-
ALP off	↓	↓	-	-
ILP on	-	-	↑	↑
ILP off	-	↓	-	-
% Active on	-	-	-	-
% Active off	↓	↓	-	↓

NAc core: nucleus accumbens core; DStr: dorsal striatum; mPFC: medial prefrontal cortex; OLPs: observing lever presses; EOLPs: extra observing lever presses; ALP on: active lever presses, light on; ALP off: active lever presses, light off; ILP on: inactive lever presses, light on; ILP off: inactive lever presses, light off; -: no significant difference relative to Shams; ↓: significant decrease relative to Shams; ↑: significant increase relative to Shams; +: significant increase relative to recovery baseline.

## Discussion

Selective excitotoxic damage to the mPFC significantly increased functional and non-functional checking and reduced discrimination accuracy during light information periods. NAc core lesions significantly increased both functional and non-functional checking, as well as transiently impairing accuracy during periods without information. DStr lesions led to a substantial reduction in instrumental responding, producing profound changes in task performance.

### Neural substrates of checking

#### mPFC and NAc core implicated in functional checking behaviour

The finding that both mPFC- and NAc core-lesioned rats increased functional checking behaviour suggests that these regions, because of their known anatomical interconnectivity, form a functional PFC–striatal circuit that is critical for the control of checking. The dorsal mPFC projects to both the NAc core and the medial DStr (Heidbreder and Groenewegen, 2003), and it is possible that both sectors of the striatum are implicated in checking. The present findings are consistent with those of ‘open-field’ checking (Szechtman et al., 1998), in which the NAc core appears to exert inhibitory control over certain components of compulsive checking (Ballester González et al., 2015; Dvorkin et al., 2010; Tucci et al., 2014). In the case of NAc core lesions, there were also a later developing increase in non-functional checking which may relate to models of OCD. For mPFC lesions, lesioned rats did exhibit increased mean levels of non-functional checking (Figure 5(f)), although the high variability in this measure precluded attainment of statistical significance. Overall, these findings support that NAc core-mPFC circuitry may contribute to maladaptive non-functional checking behaviour, with a possible focus on the nucleus accumbens.

#### DStr and checking

DStr-lesioned rats, unlike NAc core-lesioned rats, were not different from controls in their rate of checking. However, the large size of these DStr lesions, which encompassed both the medial and lateral striatum, makes interpretation of the results less straightforward, given the substantial evidence of functional heterogeneity between striatal subregions (Devan et al., 2011; White, 2009). Analysis of the data indicates that although DStr lesions did not increase or decrease checking numerically, they did substantially reduce instrumental responding, and consequently, the ratio of checking to instrumental responding was greatly increased. Thus, DStr lesions produced a qualitatively similar effect on checking to that of NAc core lesions – although in the latter, a weaker reduction in instrumental responding was accompanied by a genuine increase in checking. Therefore, although superficially different, both NAc core and DStr lesions induced a similar behavioural profile in the ORT.

### Theories that might explain increased functional checking

#### Conditioned reinforcement versus information-seeking

Two key hypotheses exist regarding the maintenance of observing or checking. The conditioned reinforcement hypothesis posits that discriminative stimuli maintain observing responses because these stimuli are associated with primary reinforcement (Dinsmoor, 1983). By contrast, the information or uncertainty reduction hypothesis argues that observing is maintained because it predicts the availability and non-availability of reinforcement (Berlyne, 1960). The profile of ORT performance supports the hypothesis that rather than responding for conditioned reinforcement, rats may be checking for information (potentially to reduce uncertainty) about the location of the active (i.e. currently rewarded) lever, consistent with Berlyne’s information-seeking hypothesis of observing. First, checking increased during periods of reward uncertainty or reward omission, when rats were potentially no longer receiving expected feedback for correct responses. Thus, rats had learned the meaning of the observing light but, under baseline task conditions, were choosing alternative strategies. Second, lesions of the NAc core have been shown to reduce the ability of a food-associated conditioned reinforcer to support the acquisition of a new instrumental response (Parkinson et al., 1999). Had the observing light acquired general affective value (through being associated with food availability), then NAc core lesions should have abolished, or at least reduced, checking, but increases were in fact observed. Therefore, the evidence is in favour of functional checking representing information-seeking and uncertainty reduction in rodents.

#### Deficient inhibitory response control

Increased functional checking could also possibly result from enhanced exploration or greater impulsivity, perhaps due to failures of behavioural inhibition mechanisms leading to hyperactivity (Maldonado-Irizarry and Kelley, 1994; Wu et al., 1993) or impulsivity (Dalley et al., 2011). However, increased checking was unlikely to reflect a generalised increase in responding, since in contrast to the increase in checking, NAc core lesions reduced instrumental responding for food and the NAc core group was not different from controls in a test of locomotor activity. It is also unlikely that increased checking in mPFC-lesioned rats was the result of generalised hyperactivity as lesioned rats did not make more instrumental responses during periods without information compared with controls.

### Increased checking is functional, arising from impaired discrimination of reward contingencies

NAc core lesions impaired rats’ ability to discriminate without information (when the active lever light was unlit). Therefore, the increased checking produced by these lesions may have arisen to compensate for this deficit by providing additional discriminatory information and is in fact fully functional. Optimal ORT performance requires rats to retrieve information about the likely location of the active lever in the absence of the exteroceptive visual cue (i.e. lever light). Therefore, they must use internally generated cues, including recent experience of reinforcement contingencies (spatio-temporal associations with reward) to guide response choice. Such a failure of discrimination performance is consistent with impairments following NAc core lesions in several studies of spatial discrimination learning (Annett et al., 1989; Klein et al., 2004; Schacter et al., 1989; Seamans and Phillips, 1994; Smith-Roe et al., 1999), although some of those studies found effects in the nucleus accumbens shell region rather than the core.

The DStr lesion produced a similar profile to the NAc core lesion in terms of impaired discrimination and preserved checking behaviour but the deficit in the uncued instrumental discrimination in the DStr group was very large compared to that seen following NAc core lesions, suggesting that the DStr group failed to compensate for this discrimination incapacity by elevating functional checking behaviour. Alternatively, high levels of motor output were disproportionately reduced by the DStr lesion, preventing any potential compensatory increases in observing.

### Effects of mPFC and striatal lesions on instrumental responding

The reduction in instrumental responding for food following NAc core lesions is unlikely to reflect impaired motor output (see above) or motivational impairment (Cardinal et al., 2002; Parkinson et al., 2000), given the intact approach responses to the active lever when lit and the unimpaired progressive ratio performance in these animals (see Supplementary Material). The impaired spatial discrimination of reinforcement contingencies may reflect specific impairments in spatial processing or in the use of interoceptive discriminative cues in relationship to reward. Thus, they may arise because of a failure of working memory for recent reward outcomes following responding in the absence of the explicit light cue.

The extensive nature of the DStr lesion makes it likely that several processes contributing to the maintenance and discrimination of instrumental responding were impaired. Not only were these DStr-lesioned rats less active but they also had reduced breakpoints on the progressive ratio schedule (see Supplementary Material). However, a primary motivational deficit seems unlikely given their normal rates of food consumption and maintenance of body weight. These findings are consistent with an extensive literature showing specific deficits in motor and motivational function following more discrete DStr lesions (Eagle et al., 1999; Fricker et al., 1996; Pisa, 1988; Whishaw et al., 1986). It is likely that the profound instrumental discrimination impairment was caused by fundamental impairments in action-outcome processing known to be produced by selective dorsomedial striatal lesions (Yin et al., 2004, 2005, 2006), possibly in combination with spatial working memory deficits as above.

The deficit in instrumental discrimination by the mPFC-lesioned rats appeared to be relatively selective in the absence of overall reductions in instrumental responding. Thus, mPFC-lesioned rats made more presses on the ‘inactive’ lever during information periods, suggestive of either a basic impairment in memory for the rule concerning reinforcement availability, a mild attentional deficit in relation to the informative visual cue or an impairment of cognitive control. There is previous evidence of a role for the mPFC in attentional functions (Birrell and Brown, 2000; Muir et al., 1996; Ragozzino et al., 1999); however, the extended duration of the visual cue implies that the attentional load in this task was relatively small. Moreover, although the increased responding on the inactive lever during the explicit visual cue is suggestive of impaired inhibitory response control, there were no other indications of such a general executive deficit. Therefore, given the role of the mPFC, specifically the prelimbic cortex (Corbit and Balleine, 2003), in action-outcome learning, it seems likely that the present deficits in reinforcement discrimination are a consequence of impairments in this inhibitory control process. The behavioural effects of mPFC lesions appeared to show some recovery with repeated testing. However, when reinforcement was made more uncertain, some of the original increases in observing became more evident in the mPFC group (Figures 9(a)). In general, reinforcement uncertainty increased observing, consistent with the hypothesis that this behaviour arises from a need to sample information under uncertainty.

### General implications

In terms of providing an adequate model of OCD, it is important to distinguish functional from non-functional checking, the severe symptoms of OCD checking presumably relating to the former. The present study has not demonstrated unequivocal non-functional checking as the increased observing could in most instances be attributed to impairments of uncertainty processing. However, there is considerable individual variability in the proportion of functional to non-functional checking and quinpirole treatment can certainly greatly enhance the latter. Therefore, the present ORT paradigm may be useful for determining how normal information-seeking can become pathological, as expressed by non-functional checking. By implementing the ORT paradigm, we have been able in this study to begin to define the neural networks controlling normal information-seeking in the context of reward and how this potentially may lead to aberrant checking behaviour.

In summary, we have shown that damage to the NAc core and mPFC significantly increased functional checking in the ORT. This is consistent with increased checking following NAc core lesions in the ‘open-field’ model of checking (Dvorkin et al., 2010). The results imply that the NAc core and mPFC form a functional PFC–striatal circuit that is critical for the control of checking behaviour and also provide support for an information-seeking account of checking in the ORT.

## Supplementary Material

Supplementary material
