# Supplementary material for: Role of the medial prefrontal cortex and nucleus accumbens in an operant model of checking behaviour and uncertainty
Source: Brain Neurosci Adv. 2017 Sep 28;1:2398212817733403. doi: 10.1177/2398212817733403 (PMC5990926; doi:10.1177/2398212817733403)
Supplement: Supplementary material [file Supplementary_Material1.pdf]

Supplementary material

Methods

Based on the known motor and motivational functions of the dorsal striatum, several control tests were performed to assess any deficits in these functions following lesions to the NAc core and DStr.

Locomotor activity. After training on the ORT, locomotor activity was measured in all rats using 10 computerised photocell beam activity cages. The cages measured 25 cm x 40 cm x 18 cm with two photocell beams dividing the length of the cage into three equal parts. Each photocell beam was positioned 1 cm above the floor of the cage. The number of beam breaks was recorded over a 60 minute period, separated into 5 minute time bins. All animals were tested in the food-deprived state.

Food pellets intake. In order to assess alterations in primary motivation, all animals were tested for food consumption while food-deprived. The amount of pellets eaten was measured during 1 hour of free access in the home cages with a single subject present. The amount of pellets eaten (in grams) was calculated by subtracting the weight of a cup containing pellets after the 1 hour test from its initial weight.

Progressive ratio. Animals were tested under a progressive ratio (PR) schedule of reinforcement to measure the breakpoint, defined as the last ratio completed by the animal. The PR paradigm was selected because PR breakpoints are believed to reflect the amount of effort an animal will exert to obtain a reinforcer (56). Animals were reinforced for lever pressing under an arithmetically increasing FR schedule in steps of 1 (i.e., 1, 2, 3, 4...). The lever press that completed each FR

within the PR schedule was reinforced with a single pellet. The duration of the progressive ratio test was 60 minutes. During the session, breakpoint was recorded.

Results

Performance during the post-operative test period. Effects on discrimination. Light on. NAc core lesions had no significant effect on discrimination during information periods. Comparison of the percentage of active versus inactive lever presses revealed that all groups made a greater percentage of active lever presses compared with inactive lever presses both pre-operatively and during each of the post-surgery phases (active vs inactive, main effect, all blocks, $p \leq .05$) (Figure S2 and Table S2).

DStr lesions had no significant effect on discrimination during information periods. Comparison of the percentage of active versus inactive lever presses revealed that all groups made a greater percentage of active lever presses compared with inactive lever presses both pre-operatively and during each of the post-surgery blocks (active vs inactive, main effect, all blocks, $p \leq .05$) (Figure S3 and Table S3).

Light off. (a) NAc core (Figure S2). Pre-operatively, all groups made a greater percentage of active lever presses compared with inactive lever presses (active vs inactive, main effect, $F_{1,15} = 171.389$, $p < .0001$) (Figure S2(b) and Table S2); and this effect did not interact significantly with lesion (active vs inactive x lesion, $F < 1$). In contrast, during the post-surgical period, NAc core lesions had a significant effect on the balance between active and inactive lever presses (active vs inactive x lesion, block 1, $F_{1,15} = 9.05$, $p < .009$; block 2, $F_{1,15} = 5.717$, $p < .03$) (Figure S2(d) and (f)).

One-way ANOVA confirmed the observation that during the post-surgical period, the NAc core group made a smaller percentage of active lever presses relative to controls and a greater percentage of inactive lever presses relative to controls (main effect, all *F*s > 5.717, $p < .05$).

(b) DStr (Figure S3). Pre-operatively, all groups made a greater percentage of active lever presses compared with inactive lever presses (active vs inactive, main effect, $F_{1,14} = 239.07$, $p < .0001$) (Figure S3b); and this effect did not interact significantly with lesion (active vs inactive x lesion, $F < 1$). In contrast, during the post-surgical period, DStr lesions had a significant effect on the balance between active and inactive lever presses (active vs inactive x lesion, block 1, $F_{1,14} = 72.168$, $p < .0001$; block 2, $F_{1,14} = 50.501$, $p < .0001$; block 3, $F_{1,14} = 60.48$, $p < .0001$; block 4, $F_{1,14} = 28.173$, $p < .0001$) (Figure S3(d), (f), (h), (j), (l)). One-way ANOVA confirmed the observation that during the post-surgical period, the DStr group made a smaller percentage of active lever presses relative to controls and a greater percentage of inactive lever presses relative to controls (main effect, all *F*s > 28.173, $p < .05$).

Unpredicted reinforcer omission. Following post-surgical testing, rats completed one omission-of-reinforcer (Ext) session and five (Experiment 1) or three (Experiment 2) recovery baseline sessions under the standard schedule of the task. The Ext session was compared with block 4 of post-surgical testing, which served as the new baseline for the following analyses.

Effects on checking. Functional checking (OLPs). Experiment 1: (a) NAc core.

When reinforcer was omitted, there was an increase in checking relative to baseline (Baseline vs Ext, main effect: $F_{1,15} = 16.742$, $p = .003$) (Figure S8(b)); however, this effect did not interact significantly with lesion (Baseline-Ext x lesion, $F < 1$). The rate of checking returned to pre-Ext levels by RB5 (Baseline vs RB5, main effect, $p > .05$).

(b) DStr. When reinforcer was omitted, DStr-lesion rats did not increase checking behaviour (Baseline-Ext x lesion, $F_{1,14} = 4.779$, $p = .046$) (Figure S8(c)). Further analysis revealed that whereas control rats increased their rate of checking relative to baseline (Baseline vs Ext, main effect, $F_{1,14} = 12.437$, $p = .006$), DStr rats did not (Baseline vs Ext, main effect, $F < 1$).

Experiment 2: When reinforcer was omitted, there was an increase in checking relative to Baseline (Baseline vs Ext, main effect: $F_{1,24} = 66.631$, $p < .0001$) (Figure S8(a)); however, this effect did not interact significantly with lesion (Baseline-Ext x lesion, $F < 1$). The rate of checking returned to pre-Ext levels during recovery baseline session 1 (RB1) (Baseline vs RB1, main effect, $p > .05$) and was lower than pre-Ext levels by RB3 (Baseline vs RB3, main effect, $p = .002$).

Non-functional checking (EOLPs). Experiment 1: (a) NAc core. When reinforcer was omitted, there was an increase in non-functional checking relative to baseline (Baseline vs Ext, main effect: $F_{1,15} = 5.022$, $p = .041$) (Figure S8(e)); however, this effect did not interact significantly with lesion (Baseline-Ext x lesion, $F < 1$). The rate of non-functional checking returned to pre-Ext levels by RB5 (Baseline vs RB5, main effect, $p > .05$).

(b) DStr. There was no effect of reinforcer omission on the rate of non-functional checking in any of the groups (Baseline vs Ext, main effect: $F < 1$) (Figure S8(f)).

Experiment 2: When reinforcer was omitted, there was an increase in non-functional checking relative to baseline (Baseline vs Ext, main effect: $F_{1,24} = 18.642$, $p < .0001$) (Figure S8(d)); however, this effect did not interact significantly with lesion (Baseline-Ext x lesion, $F < 1$). The rate of non-functional checking returned to pre-Ext levels by RB1 (Baseline vs RB1, main effect, $p > .05$) and was lower than pre-Ext levels by RB3 (Baseline vs RB3, main effect, $p = .031$).

Effects on active lever presses. Experiment 1: (a) NAc core. During reinforcer omission, there was a significant reduction in the rate of active lever presses when the active lever light was unlit (Baseline vs Ext, main effect: $F_{1,15} = 7.979$, $p = .013$) (Figure S8(h)); however, this effect did not interact significantly with lesion (Baseline-Ext x lesion, $F_{1,15} = 1.914$, $p = .187$, NS). The rate of active lever presses returned to pre-Ext levels by RB5 (Baseline vs RB5, main effect, $p > .05$). There was no change in the rate of active lever presses when the active lever light was lit (Baseline vs Ext, main effect, $F < 1$) (Figure not shown).

(b) DStr. When reinforcer was omitted, DStr-lesion rats did not reduce their rate of active lever presses (Baseline-Ext x lesion, $F_{1,14} = 11.128$, $p = .005$) (Figure S8(i)). Further analysis revealed that whereas control rats reduced their rate of active lever

presses relative to baseline (Baseline vs Ext, main effect, $F_{1,9} = 22.799$, $p = .001$), DStr rats did not (Baseline vs Ext, main effect, $F < 1$).

Experiment 2: During reinforcer omission, there was a significant reduction in the rate of active lever presses, both when the active lever light was lit (Baseline vs Ext, main effect: $F_{1,22} = 6.224$, $p = .021$) (Figure not shown) and when it was unlit (Baseline vs Ext, main effect: $F_{1,24} = 88.527$, $p < .0001$) (Figure S8(g)); however, this effect did not interact significantly with lesion (Baseline-Ext x lesion, $F < 1$). The rate of active lever presses returned to pre-Ext levels by RB3 (Baseline vs RB3, main effect, $p > .05$).

Control tests. Locomotor activity. A repeated measures ANOVA revealed a main effect of lesion ($F_{2,20} = 3.970$, $p = .035$), together with a main effect of time bin ($F_{7.112,142.234} = 18.122$, $\tilde{\varepsilon} = 0.647$, $p = .0001$; *post hoc*: time bins 2-12, $p < .05$), reflecting habituation to the novel environment, with no interaction ($F < 1$) (Figure S9(a)). The *post hoc* comparisons confirmed the observation that rats with DStr lesions showed lower levels of spontaneous locomotor activity compared with control rats ($p = .037$) and NAc core-lesion rats ($p = .014$).

Food consumption test. One-way ANOVA showed no differences in the amount of food (in g) that NAc core, DStr and control rats ate in a 60 minute test where pellets were freely available ($F < 1$) (Figure S9(b)). Mean grams of pellets eaten ± SEM: Sham, 14.55 ± 1.78; NAc core, 13.04 ± 2.83; DStr, 14.28 ± 1.1.

Progressive ratio test. An analysis of breakpoint showed a significant effect of lesion ($F_{2,22}$ = 6.624, p = .006) (Figure S9 (c)). The *post hoc* comparisons confirmed the observation that the DStr group had a lower breakpoint than both sham (p = .003) and NAc core (p = .007) subjects under a progressive ratio schedule of responding. Breakpoint ± SEM: Sham, 76.7 ± 4.44; NAc core, 74.9 ± 8.58; DStr, 46.33 ± 6.73.

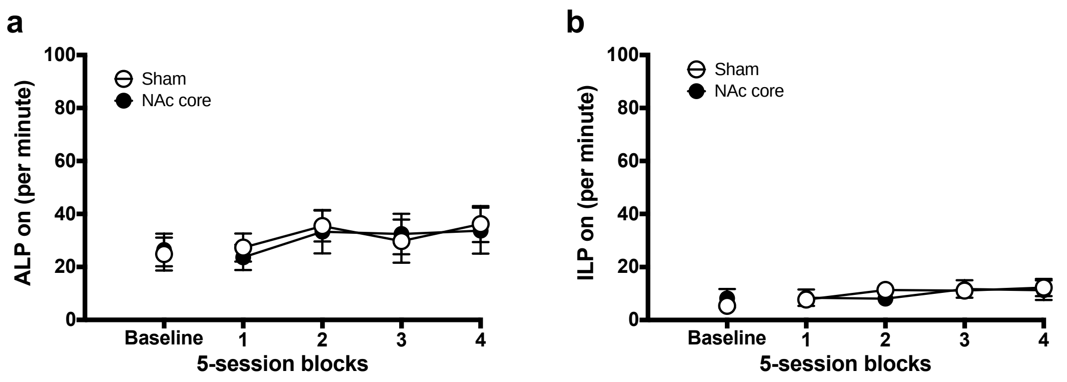

Figure S1. The effect of NAc core lesions on instrumental responding. Figures show pre-surgery baseline session (3 sessions) and 20 post-surgical sessions for NAc core-lesion rats and sham-operated controls. (a) ALP on (b) ILP on.

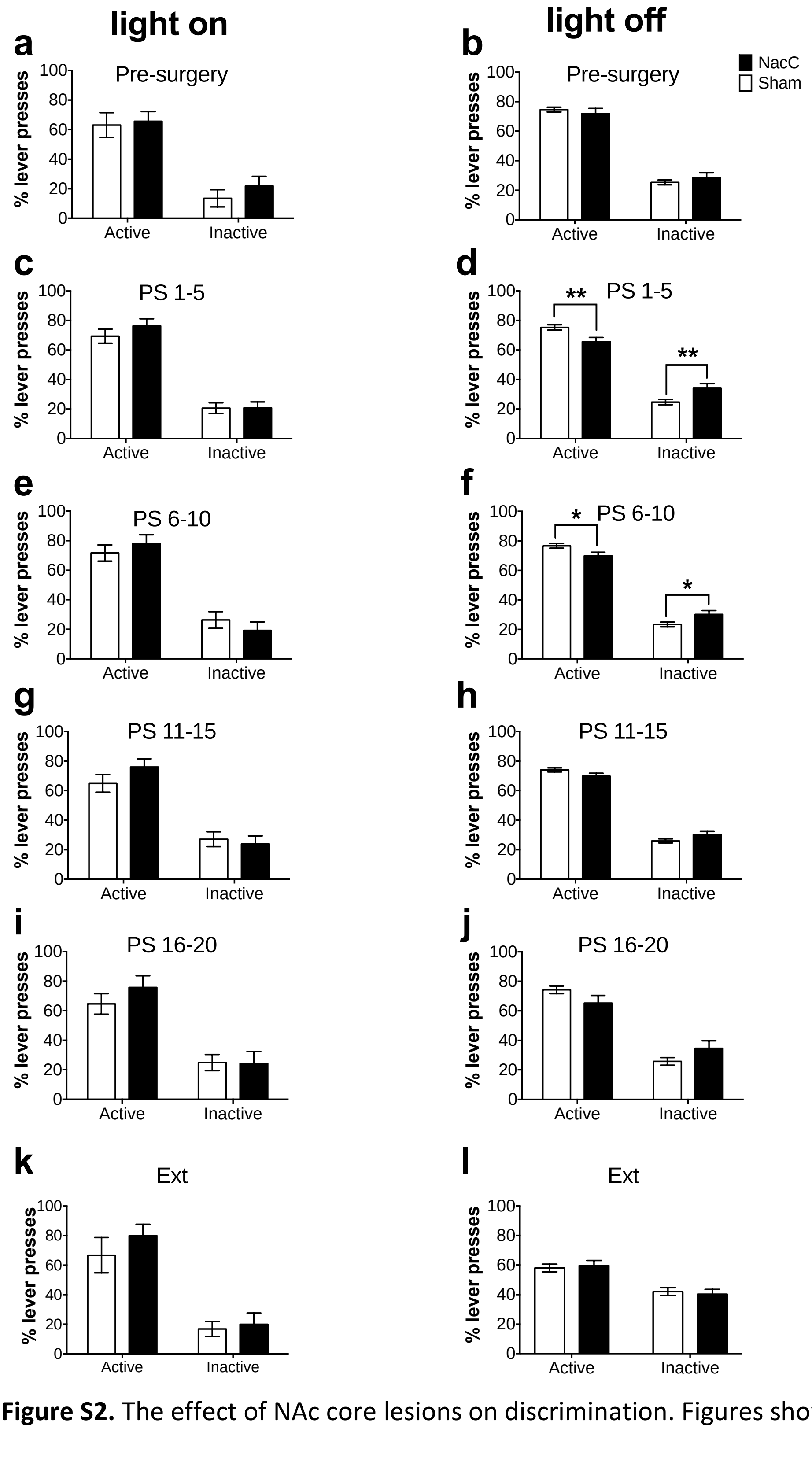

Figure S2. The effect of NAc core lesions on discrimination. Figures show the percentage of lever presses, when the light was lit (left) and when the light was unlit (right), for NAc core-lesion rats and controls. Results are shown as a mean of 3-

session blocks (pre-surgery), 5-session blocks (PS 1-5, 6-10, 11-15, 16-20) and a single omission-of-reinforcer session (Ext). * Denotes a significant difference between the groups. Significance is denoted as follows: *$p < 0.05$, **$p < 0.01$ between groups.

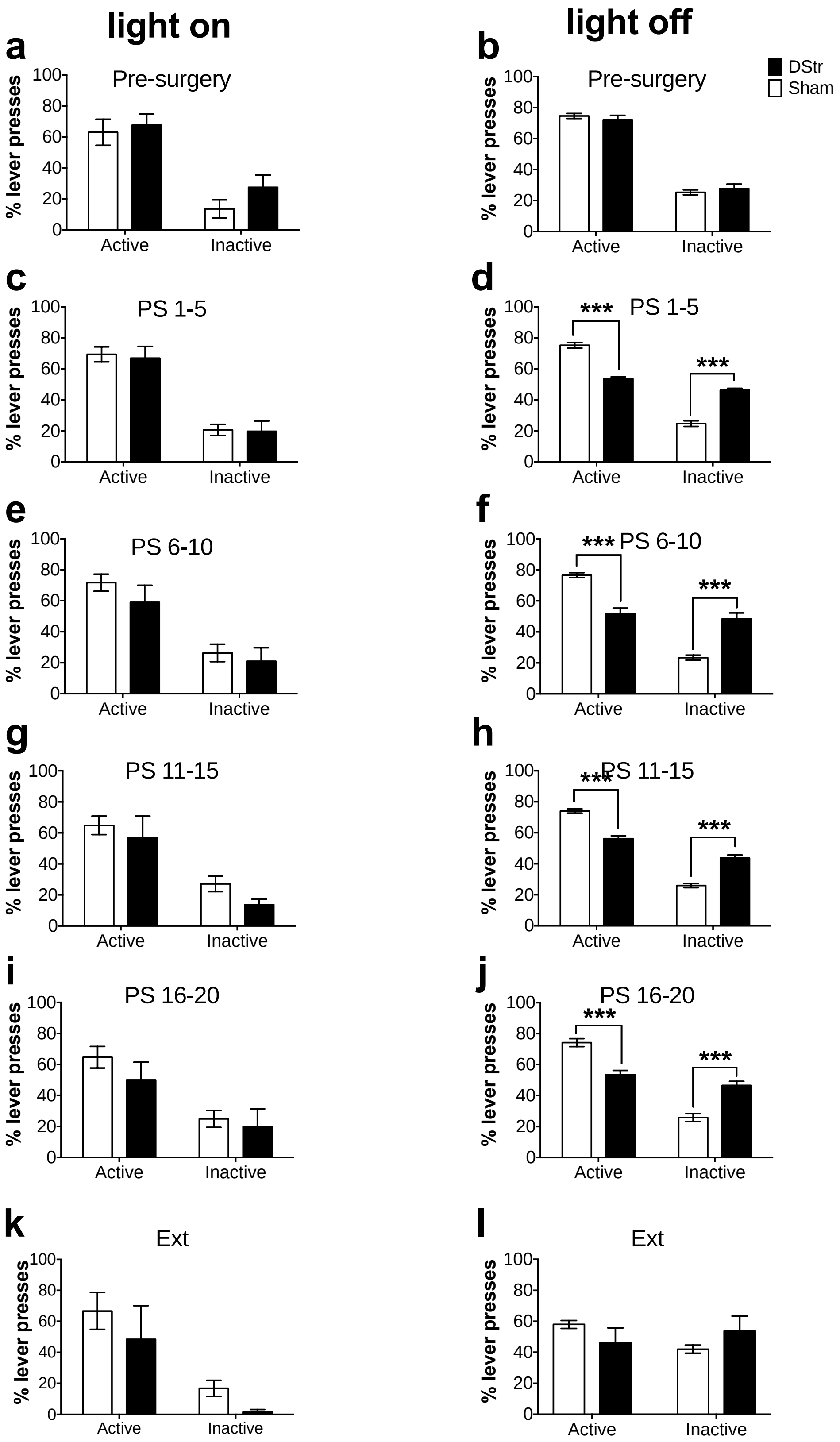

Figure S3. The effect of DStr lesions on discrimination. Figures show the percentage of lever presses, when the light was lit (left) and when the light was unlit (right), for DStr-lesion rats and controls. Results are shown as a mean of 3-session blocks (pre-

surgery), 5-session blocks (PS 1-5, 6-10, 11-15, 16-20) and a single omission-of-reinforcer session (Ext). Significance is denoted as follows: *$p < 0.05$, **$p < 0.01$, ***$p < 0.001$ between groups.

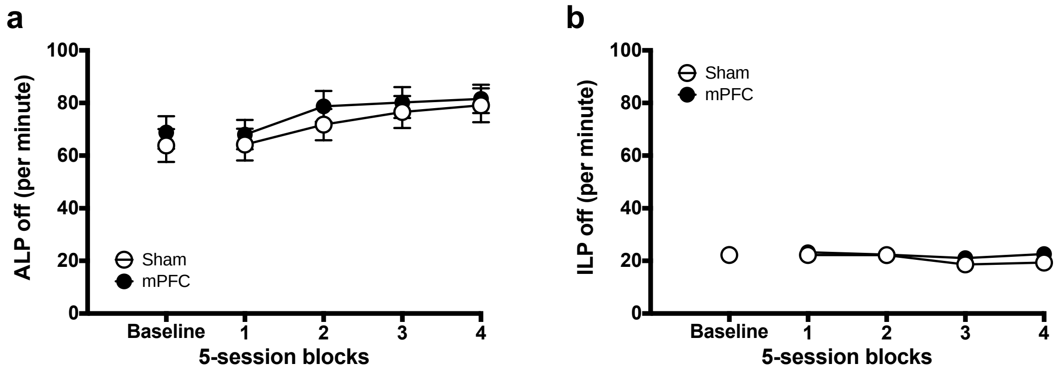

Figure S4. The effect of mPFC lesions on instrumental responding. Figures show pre-surgery baseline sessions (3 sessions) and 20 post-surgical sessions for mPFC-lesion rats and sham-operated controls. (a) ALP off (b) ILP off.

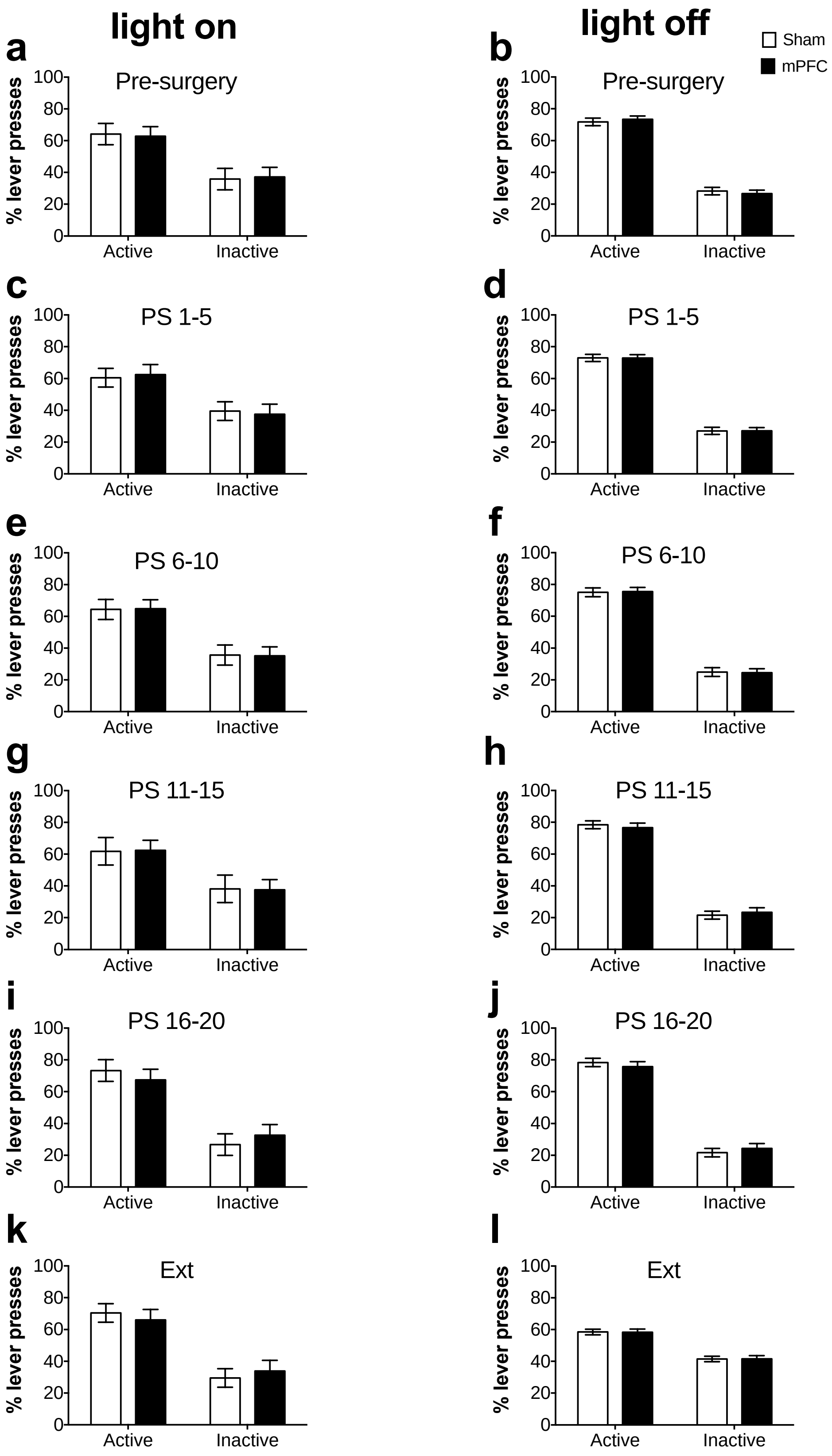

Figure S5. The effect of mPFC lesions on discrimination. Figures show the percentage of lever presses, when the light was lit (left) and when the light was unlit (right), for mPFC-lesion rats and controls. Results are shown as a mean of 3-session blocks (pre-

surgery), 5-session blocks (PS 1-5, 6-10, 11-15, 16-20) and a single omission-of-reinforcer session (Ext).

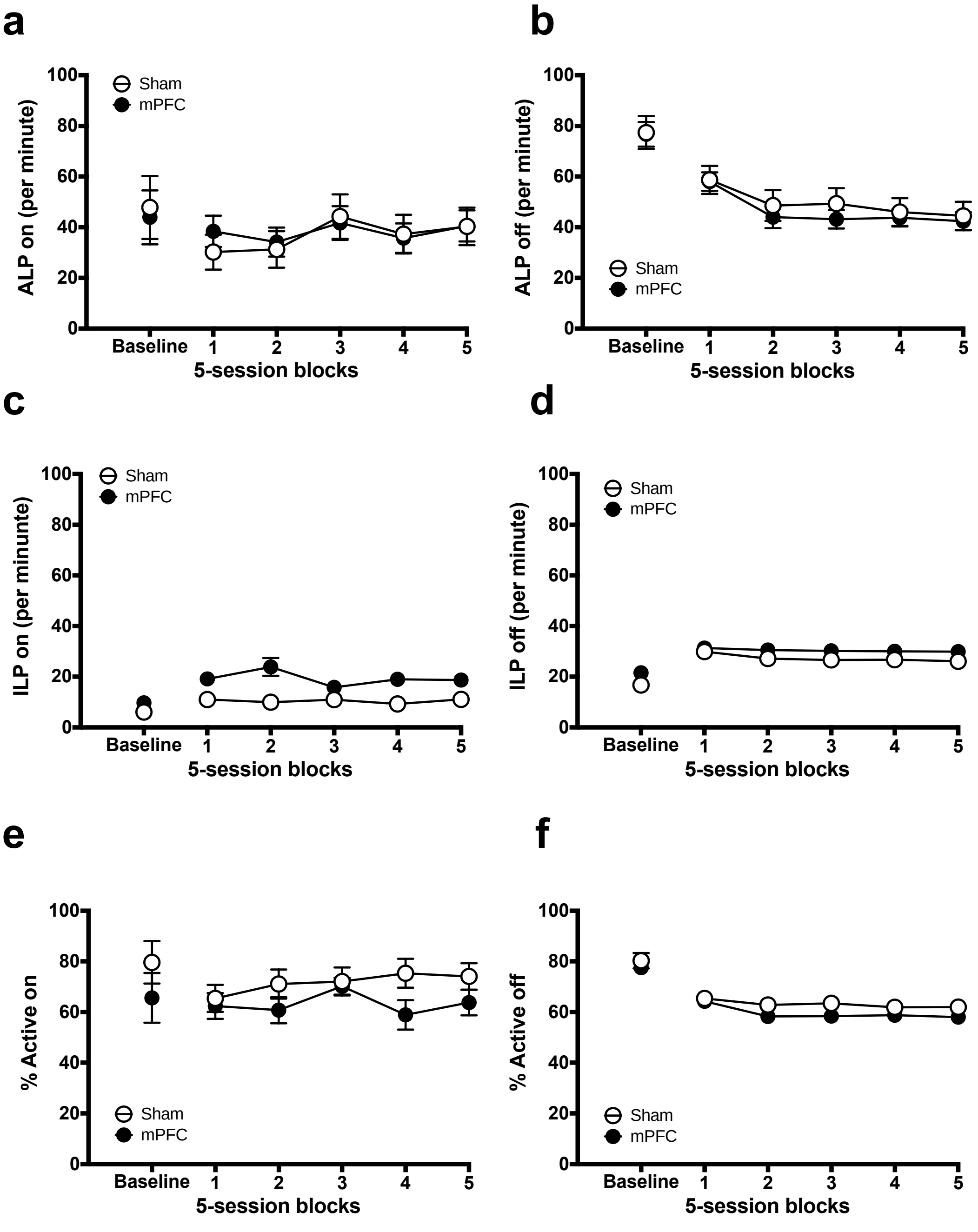

Figure S6. Lack of effect of uncertainty on measures of instrumental responding. Figures show baseline session (3 sessions under the standard schedule of the task) and 25 sessions of uncertainty for mPFC-lesion rats and sham-operated controls. (a) ALP on (b) ALP off (c) ILP on (d) ILP off (e) % Active on (f) % Active off.

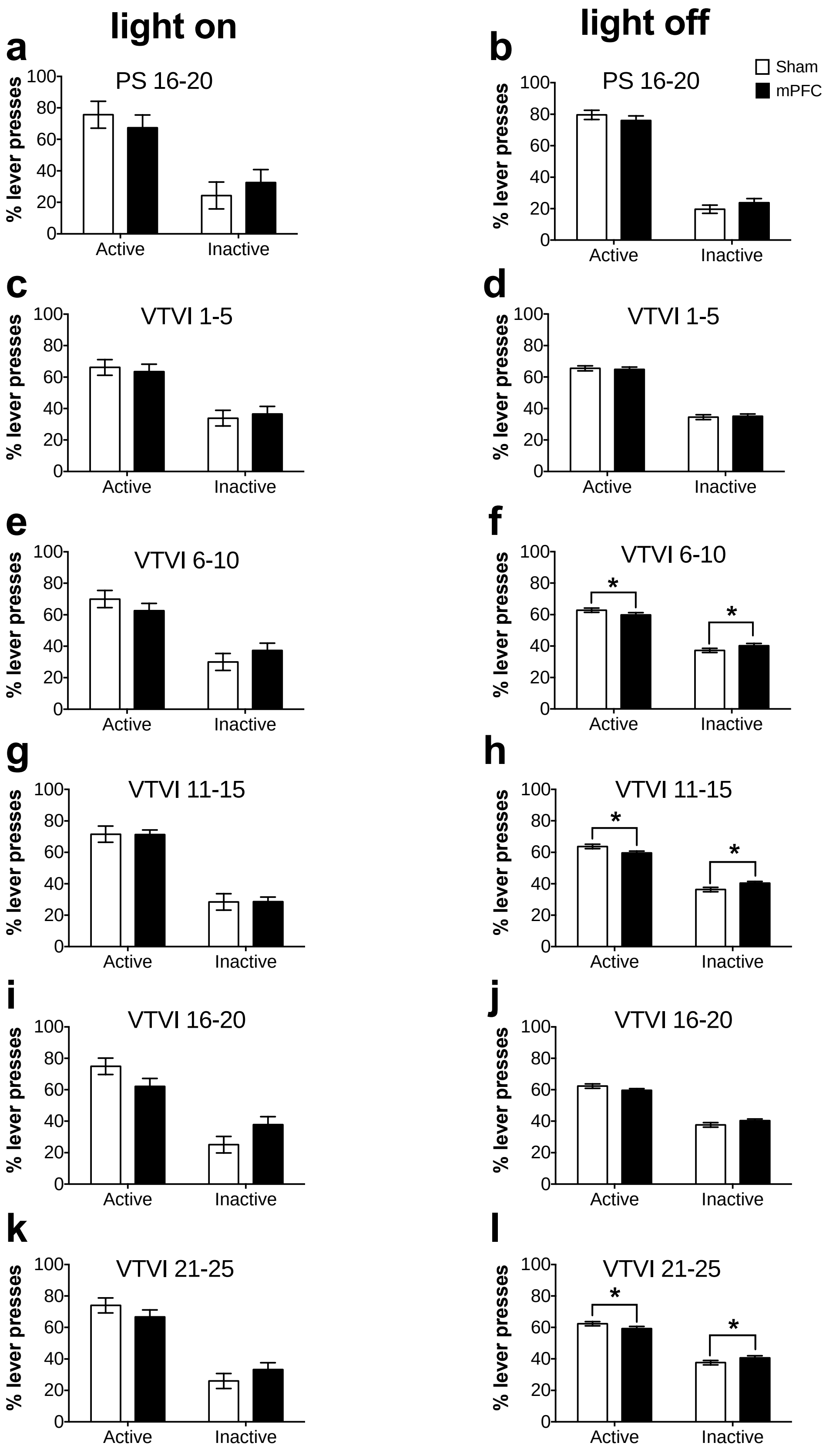

light on
light off
a
PS 16-20
b
PS 16-20
Sham
mPFC
c
VTVI 1-5
d
VTVI 1-5
e
VTVI 6-10
f
VTVI 6-10
g
VTVI 11-15
h
VTVI 11-15
i
VTVI 16-20
j
VTVI 16-20
k
VTVI 21-25
l
VTVI 21-25
% lever presses
Active
Inactive

Figure S7. The effect of uncertainty on discrimination. Figures show the percentage of lever presses, when the light was lit (left) and when the light was unlit (right), for mPFC-lesion rats and controls. Results are shown as a mean of 5-session blocks. Significance is denoted as follows: *$p < 0.05$ between groups.

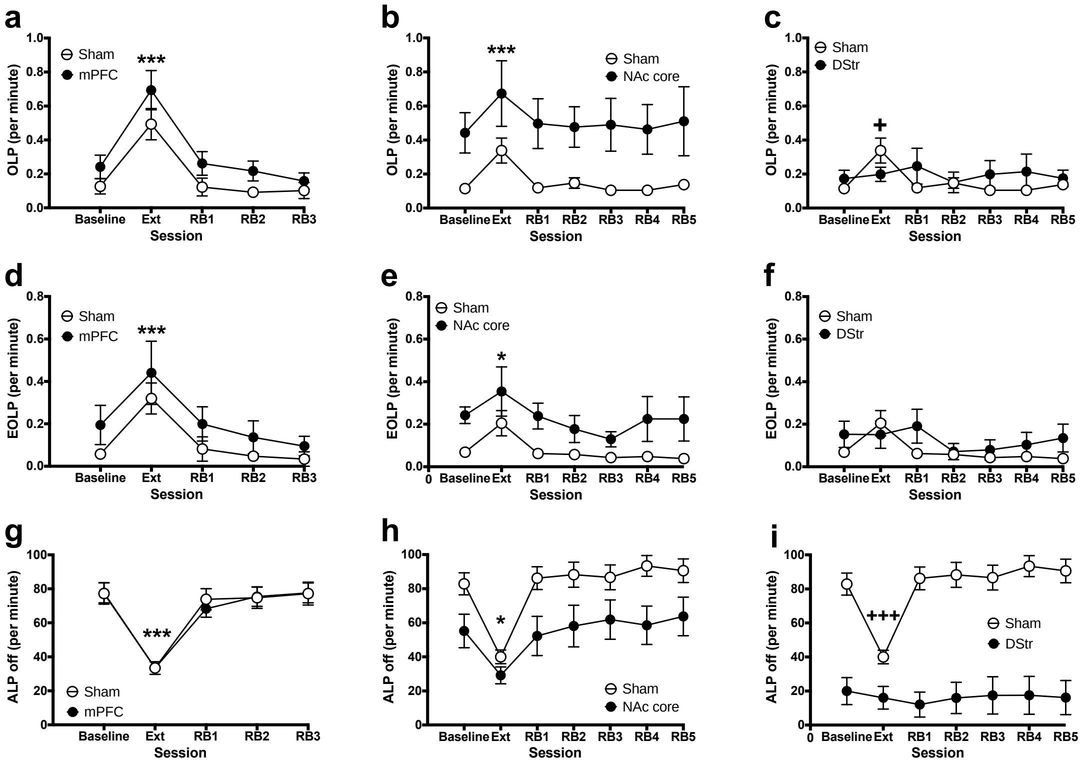

Figure S8. The effects of unpredicted omission-of-reinforcer. Top: Functional checking (OLPs) in (a) mPFC- (b) NAc core- (c) DStr-lesion rats. Middle: Non-functional checking (EOLPs) in (d) mPFC- (e) NAc core- (f) DStr-lesion rats. Bottom: ALP off in (g) mPFC- (h) NAc core- (i) DStr-lesion rats. Significance is denoted as follows: *$p < 0.05$, *** $p < 0.001$ versus baseline (main effect); +$p < 0.05$, +++$p < 0.001$ versus baseline in the Sham group (interaction).

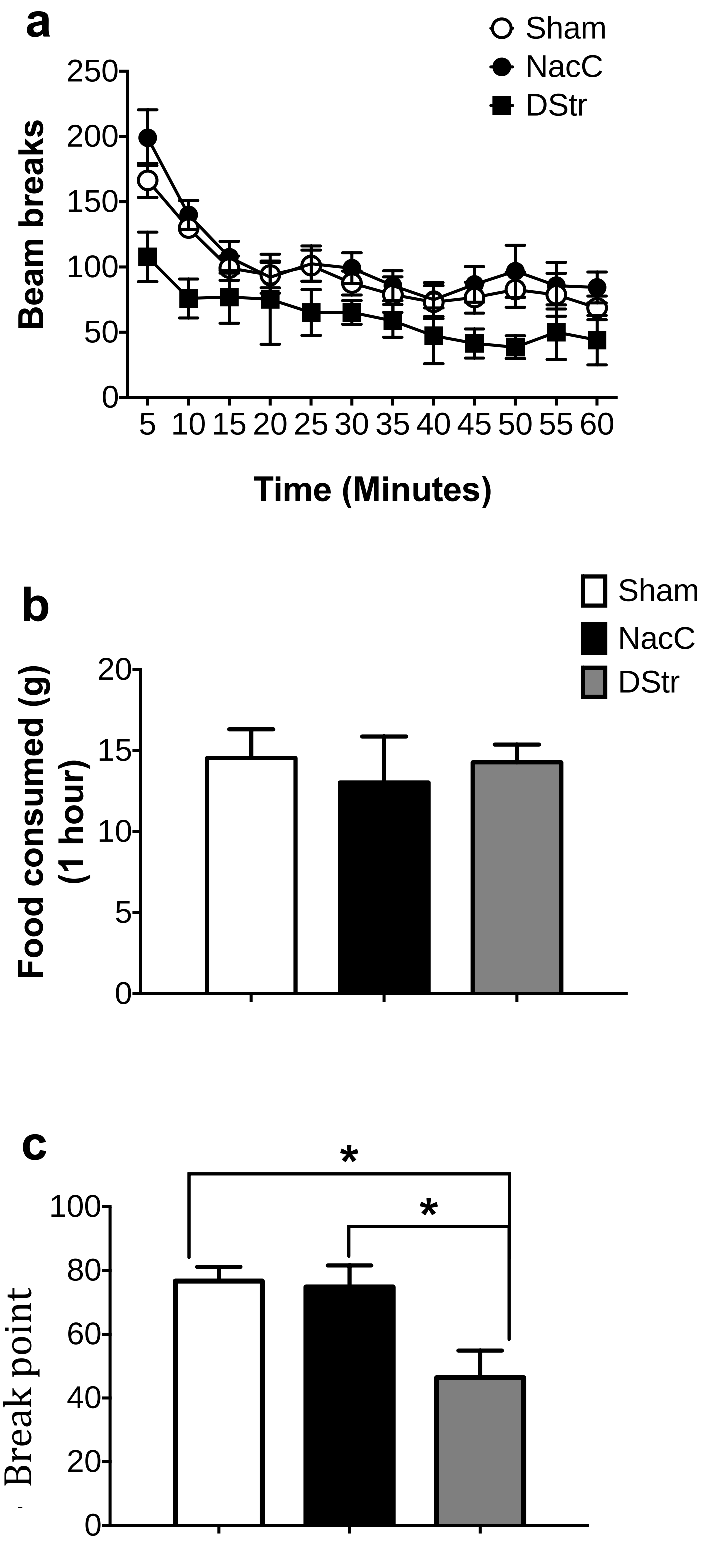

Figure S9. (a) Locomotor activity. Controls, open circles; DStr, closed squares; NAc core, closed circles (b) Food consumed in a 1 hour test session. (c) Breakpoint in a

progressive ratio schedule of reinforcement. Controls, open bars; DStr, grey bars; NAc core, black bars. * Denotes a significant difference between groups.

Measure	PS1-5		PS6-10		PS11-15		PS16-20		
	Pre-Post block x Lesion Interaction	Post Group Effect	Pre-Post block x Lesion Interaction	Post Group Effect	Pre-Post block x Lesion Interaction	Post Group Effect	Pre-Post block x Lesion Interaction	Post Group Effect	
**ALP on**	*F* < 1	*F* < 1	*F* < 1	*F* < 1	*F* < 1	*F* < 1	*F* < 1	*F* < 1	
**ILP on**	*F* < 1	*F* < 1	$F_{1,15}$ = 2.126, *p* = .165	*F* < 1	*F* < 1	*F* < 1	*F* < 1	*F* < 1	
**ILP off**	*F* < 1	**$F_{1,16}$ = 6.848, *p* = .09 $**	$F_{1,15}$ = 1.104, *p* = .31	$F_{1,16}$ = 1.172, *p* = .296	**$F_{1,15}$ = 6.027, *p* = .027** *	**$F_{1,16}$ = 6.848, *p* = .019 $**	$F_{1,15}$ = 1.777, *p* = .202	$F_{1,16}$ = 1.283, *p* = .275	* Significant increase in controls relative to baseline<br><br>$ NAc core < controls
**% Active on**	*F* < 1	*F* < 1	*F* < 1	*F* < 1	*F* < 1	$F_{1,16}$ = 1.722, *p* = .209	*F* < 1	$F_{1,16}$ = 1.337, *p* = .266	
**% Active off**	**$F_{1,15}$ = 9.364, *p* = .008 ***	**$F_{1,16}$ = 9.05, *p* = .009 $**	$F_{1,15}$ = 1.477, *p* = .243	**$F_{1,16}$ = 5.717, *p* = .03 $**	*F* < 1	$F_{1,16}$ = 3.271, *p* = .091	$F_{1,15}$ = 2.949, *p* = .106	$F_{1,16}$ = 2.939, *p* = .107	* Significant reduction in NAc core rats relative to baseline<br><br>$ NAc core < controls

Table S1. The effect of NAc core lesions on instrumental responding. Table shows analyses for 20 post-surgical sessions (5-session blocks) for measures: ALP on, ILP on, ILP off, % Active on, % Active off. Significant analyses in bold. * Denotes a significant interaction. $ Denotes a significant effect of Lesion. Pre, pre-surgical baseline. Post, post-surgical sessions. See also Figure S1.

Measure	Baseline		PS1-5		PS6-10		PS11-15		PS16-20		Ext	
	Active-Inactive Main effect	Active-Inactive x Lesion Interaction	Active-Inactive Main effect	Active-Inactive x Lesion Interaction	Active-Inactive Main effect	Active-Inactive x Lesion Interaction	Active-Inactive Main effect	Active-Inactive x Lesion Interaction	Active-Inactive Main effect	Active-Inactive x Lesion Interaction	Active-Inactive Main effect	Active-Inactive x Lesion Interaction
**Light on**	**$F_{1,15}$ = 29.227, p < .0001**	F < 1	**$F_{1,15}$ = 87.166, p < .0001**	F < 1	**$F_{1,15}$ = 40.87, p < .0001**	F < 1	**$F_{1,15}$ = 34.283, p < .0001**	F < 1	**$F_{1,15}$ = 22.05, p < .0001**	F < 1	**$F_{1,15}$ = 26.018, p ≤ .0001**	F < 1
**Light off**	**$F_{1,15}$ = 171.389, p < .0001**	F < 1	**$F_{1,15}$ = 162.337, p < .0001**	**$F_{1,15}$ = 9.05, p = .009 ***	**$F_{1,15}$ = 262.995, p < .0001**	**$F_{1,15}$ = 5.717, p = .03 ***	**$F_{1,15}$ = 340.357, p < .0001**	$F_{1,15}$ = 3.271, p = .091	**$F_{1,15}$ = 58.09, p < .0001**	$F_{1,15}$ = 2.939, p = .107	**$F_{1,15}$ = 18.339, p = .001**	F < 1

Table S2. Effects of NAc core lesions on discrimination. Table shows analyses for pre-surgery baseline (3-session blocks); 20 post-surgical sessions (5-session blocks); one omission-of-reinforcer session (Ext) for measures: Light on, Light off. Significant analyses in bold. See also Figure S2.

Measure	Baseline		PS1-5		PS6-10		PS11-15		PS16-20		Ext	
	Active-Inactive Main effect	Active-Inactive x Lesion Interaction	Active-Inactive Main effect	Active-Inactive x Lesion Interaction	Active-Inactive Main effect	Active-Inactive x Lesion Interaction	Active-Inactive Main effect	Active-Inactive x Lesion Interaction	Active-Inactive Main effect	Active-Inactive x Lesion Interaction	Active-Inactive Main effect	Active-Inactive x Lesion Interaction
**Light on**	**$F_{1,14}$ = 22.801, *p* = .0002**	$F < 1$	**$F_{1,14}$ = 61.142, *p* < .0001**	$F < 1$	**$F_{1,14}$ = 19.832, *p* = .001**	$F < 1$	**$F_{1,14}$ = 25.648, *p* = .00017**	$F < 1$	**$F_{1,14}$ = 14.433, *p* = .002**	$F < 1$	**$F_{1,14}$ = 15.168, *p* = .002**	$F < 1$
**Light off**	**$F_{1,14}$ = 239.07, *p* < .0001**	$F < 1$	**$F_{1,14}$ = 129.146, *p* < .0001**	**$F_{1,14}$ = 72.168, *p* < .0001**	**$F_{1,14}$ = 64.666, *p* < .0001**	**$F_{1,14}$ = 50.501, *p* < .0001**	**$F_{1,14}$ = 173.864, *p* < .0001**	**$F_{1,14}$ = 60.48, *p* < .0001**	**$F_{1,14}$ = 50.451, *p* < .0001**	**$F_{1,14}$ = 28.173, *p* = .00011**	$F < 1$	$F_{1,14}$ = 2.197, *p* = .16

Table S3. Effects of DStr lesions on discrimination. Table shows analyses for pre-surgery baseline (3-session blocks); 20 post-surgical sessions (5-session blocks); one omission-of-reinforcer session (Ext) for % lever presses during Light on, Light off. Significant analyses in bold. Active, % active lever presses; % Inactive, inactive lever presses. See also Figure S3.

Measure	PS1-5		PS6-10		PS11-15		PS16-20	
	Pre-Post block x Lesion Interaction	Post Group Effect	Pre-Post block x Lesion Interaction	Post Group Effect	Pre-Post block x Lesion Interaction	Post Group Effect	Pre-Post block x Lesion Interaction	Post Group Effect
**ALP on**	$F_{1,24} = 3.152$, $p = .089$	$F < 1$	$F_{1,24} = 1.039$, $p = .318$	$F < 1$	$F_{1,24} = 4.103$, $p = .054$	$F < 1$	$F_{1,23} = 1.23$, $p = .279$	$F < 1$
**ALP off**	$F < 1$	$F < 1$	$F < 1$	$F < 1$	$F < 1$	$F < 1$	$F < 1$	$F < 1$
**ILP off**	$F < 1$	$F < 1$	$F < 1$	$F < 1$	$F_{1,24} = 1.284$, $p = .268$	$F_{1,25} = 2.229$, $p = .148$	$F_{1,24} = 1.563$, $p = .223$	$F_{1,25} = 2.169$, $p = .154$
**% Active on**	$F < 1$	$F < 1$	$F < 1$	$F < 1$	$F < 1$	$F < 1$	$F < 1$	$F < 1$
**% Active off**	$F < 1$	$F < 1$	$F < 1$	$F < 1$	$F < 1$	$F < 1$	$F < 1$	$F < 1$

Table S4. The effect of mPFC lesions on instrumental responding. Table shows analyses for 20 post-surgical sessions (5-session blocks) for measures: ALP on, ILP on, ILP off, % Active on, % Active off. See also Figure S4. Pre, pre-surgical baseline. Post, post-surgical sessions.

Measure	Baseline		PS1-5		PS6-10		PS11-15		PS16-20		Ext	
	Active-Inactive Main effect	Active-Inactive x Lesion Interaction	Active-Inactive Main effect	Active-Inactive x Lesion Interaction	Active-Inactive Main effect	Active-Inactive x Lesion Interaction	Active-Inactive Main effect	Active-Inactive x Lesion Interaction	Active-Inactive Main effect	Active-Inactive x Lesion Interaction	Active-Inactive Main effect	Active-Inactive x Lesion Interaction
**Light on**	**$F_{1,24}$ = 6.831, p = .015**	$F < 1$	**$F_{1,24}$ = 7.076, p = .014**	$F < 1$	**$F_{1,24}$ = 8.257, p = .008**	$F < 1$	$F_{1,24}$ = 2.803, p = .107	$F < 1$	**$F_{1,22}$ = 15.524, p = .001**	$F < 1$	**$F_{1,23}$ = 18.471, $p \leq$ .00026**	$F < 1$
**Light off**	**$F_{1,24}$ = 160.358, $p <$ .0001**	$F < 1$	**$F_{1,24}$ = 187.984, $p <$ .0001**	$F < 1$	**$F_{1,24}$ = 195.744, $p <$ .0001**	$F < 1$	**$F_{1,24}$ = 284.987, $p <$ .0001**	$F < 1$	**$F_{1,24}$ = 227.573, $p <$ .0001**	$F < 1$	**$F_{1,24}$ = 38.967, $p <$ .0001**	$F < 1$

Table S5. The effect of mPFC lesions on discrimination. Table shows analyses for pre-surgery baseline (3-session blocks); 20 post-surgical sessions (5-session blocks); one omission-of-reinforcer session (Ext) for % lever presses during Light on, Light off. Significant analyses in bold. Active, % active lever presses; % Inactive, inactive lever presses. See also Figure S5.

Measure	VTVI1-5		VTVI6-10		VTVI11-15		VTVI16-20		VTVI21-25		
	Interaction	Group	Interaction	Group	Interaction	Group	Interaction	Group	Interaction	Group	
ALP on	*F* < 1	*F* < 1	*F* < 1	*F* < 1	*F* < 1	*F* < 1	*F* < 1	*F* < 1	*F* < 1	*F* < 1	
ALP off	*F* < 1	*F* < 1	*F* < 1	*F* < 1	*F* < 1	*F* < 1	*F* < 1	*F* < 1	*F* < 1	*F* < 1	
ILP off	$F_{1,24}$ = 1.117, *p* = .301	*F* < 1	*F* < 1	*F* < 1	*F* < 1	$F_{1,25}$ = 1.414, *p* = .246	*F* < 1	$F_{1,25}$ = 1.43, *p* = .243	*F* < 1	$F_{1,25}$ = 1.621, *p* = .215	
% Active on	*F* < 1	*F* < 1	*F* < 1	$F_{1,24}$ = 1.807, *p* = .192	*F* < 1	*F* < 1	*F* < 1	$F_{1,23}$ = 4.004, *p* = .058	*F* < 1	$F_{1,21}$ = 1.98, *p* = .175	
% Active off	*F* < 1	*F* < 1	*F* < 1	**$F_{1,25}$ = 5.289, *p* = .03***	*F* < 1	**$F_{1,25}$ = 7.273, *p* = .013 ***	*F* < 1	$F_{1,25}$ = 2.848, *p* = .104	*F* < 1	**$F_{1,25}$ = 4.531, *p* = .044***	*** mPFC < controls**

Table S6. The effect of uncertainty on instrumental responding. Table shows analyses for 25 uncertainty sessions (5-session blocks) for measures: ALP on, ALP off, ILP off, % Active on, % Active off. Significant analyses in bold. Pre, pre-surgical baseline. Post, post-surgical sessions. See also Figure S6.

Measure	Re-baseline		VTVI1-5		VTVI6-10		VTVI11-15		VTVI16-20		VTVI21-25	
	Active-Inactive Main effect	Active-Inactive x Lesion Interaction	Active-Inactive Main effect	Active-Inactive x Lesion Interaction	Active-Inactive Main effect	Active-Inactive x Lesion Interaction	Active-Inactive Main effect	Active-Inactive x Lesion Interaction	Active-Inactive Main effect	Active-Inactive x Lesion Interaction	Active-Inactive Main effect	Active-Inactive x Lesion Interaction
**Light on**	**$F_{1,20}$ = 11.689, *p* = .003**	$F_{1,20}$ = 1.123, *p* = .302	**$F_{1,23}$ = 14.174, *p* = .001**	*F* < 1	**$F_{1,23}$ = 17.18, *p* = .00039**	$F_{1,23}$ = 1.807, *p* = .192	**$F_{1,23}$ = 40.928, *p* < .0001**	*F* < 1	**$F_{1,22}$ = 17.417, *p* = .00039**	$F_{1,22}$ = 4.004, *p* = .058	**$F_{1,20}$ = 26.692, *p* < .0001**	$F_{1,20}$ = 1.98, *p* = .175
**Light off**	**$F_{1,24}$ = 271.553, *p* < .0001**	*F* < 1	**$F_{1,24}$ = 179.244, *p* < .0001**	*F* < 1	**$F_{1,24}$ = 113.439, *p* < .0001**	**$F_{1,24}$ = 5.289, *p* = .03**	**$F_{1,24}$ = 135.843, *p* < .0001**	**$F_{1,24}$ = 7.273, *p* < .013**	**$F_{1,24}$ = 123.852, *p* < .0001**	$F_{1,24}$ = 2.848, *p* = .104	**$F_{1,24}$ = 114.524, *p* < .0001**	**$F_{1,24}$ = 4.531, *p* = .044**

Table S7. Effects of uncertainty on discrimination. Table shows analyses for baseline (3-session blocks); 25 uncertainty sessions (5-session blocks) for % lever presses during Light on, Light off. Significant analyses in bold. Active, % active lever presses; % Inactive, inactive lever presses. See also Figure S7.
